# Confocal laser endomicroscopy as predictive biomarker of clinical and endoscopic efficacy of vedolizumab in ulcerative colitis: The DETECT study

**DOI:** 10.1371/journal.pone.0298313

**Published:** 2024-04-02

**Authors:** Lucille Quénéhervé, Caroline Trang-Poisson, Aurélie Fantou, Mathurin Flamant, Tony Durand, Guillaume Bouguen, Jérémy Bregeon, Thibauld Oullier, Morgane Amil, Marie Dewitte, Stéphanie Bardot, Stéphanie Blandin, Cécile Braudeau, Marie-Anne Vibet, Régis Josien, Michel Neunlist, Arnaud Bourreille

**Affiliations:** 1 Department of Gastroenterology, University Hospital of Brest, Brest, France; 2 Nantes Université, CHU Nantes, Institut des Maladies de l’Appareil Digestif (IMAD), Hépato-Gastroentérologie, Inserm CIC 1413, Nantes, France; 3 Nantes Université, CHU Nantes, CRT2I, UMR Inserm 1064, Nantes, France; 4 Nantes Université, CHU Nantes, Institut des Maladies de l’Appareil Digestif (IMAD), UMR Inserm 1235 TENS, Nantes, France; 5 Université de Rennes, CHU Rennes, Institut NUMECAN (Nutrition Metabolism and Cancer), Hepato-Gastroenterologie, Inserm CIC1414, Rennes, France; 6 CHD La Roche-Sur-Yon, Hepato-gastroentérologie, La Roche-Sur-Yon, France; 7 Nantes Université, UMS BioCore, Inserm US16—UAR CNRS 3556, Nantes, France; 8 CHU Nantes, Methodology and Biostatistics Department, Direction de la Recherche Clinique et de l’Innovation, Nantes, France; The University of Hong Kong Li Ka Shing Faculty of Medicine, HONG KONG

## Abstract

**Aims:**

In patients with ulcerative colitis (UC), no biomarker is available to help the physician to choose the most suitable biotherapy. The primary objective of this pilot study was to assess the feasibility of identification of α4β7- and TNF-expressing cells, to predict the response to vedolizumab using confocal laser endoscopy (CLE).

**Methods:**

Patients with moderate-to-severe UC, naïve of biotherapy, received vedolizumab. Clinical evaluation was performed at each infusion. Endoscopic evaluation was performed before inclusion and at week 22. Fresh colonic biopsies were stained using FITC-labelled vedolizumab and Alexa fluor-labelled adalimumab and *ex vivo* dual-band CLE images were acquired. Blood samples were collected to measure trough concentrations of vedolizumab and to determine absolute counts of T and B cells subpopulations, NK cells and monocytes.

**Results:**

Nineteen patients were enrolled in the study and received at least one dose of vedolizumab. Clinical remission and endoscopic improvement were observed in 58% of whom 5 patients (45%) had an endoscopic subscore of 0. In terms of clinical response and remission, endoscopic improvement and histologic response, FITC-conjugated vedolizumab staining tended to be higher in responder patients compared to non-responders at week 22. A threshold value of 6 positive FITC-vedolizumab staining areas detected by CLE seemed informative to discriminate the responders and non-responders. The results were similar in terms of clinical remission and endoscopic improvement with a sensitivity of 78% and a specificity of 85% (p = 0.05). Trough concentrations and blood immune cells were not associated with responses to vedolizumab.

**Conclusion:**

This pilot study demonstrate that dual-band CLE is feasible to detect α4β7- and TNF-expressing cells. Positive α4β7 staining seems to be associated with clinical and endoscopic remission in UC patients treated by anti-α4β7-integrin, subject to validation by larger-scale studies.

Clinical-trial.gov: NCT02878083

## Introduction

Biotherapies are widely used in patients with moderate to severe Ulcerative Colitis (UC) who are resistant or intolerant to conventional treatment. Most of the clinicians have the possibility to use, in such condition, anti-TNFs or vedolizumab. The choice between the different families of biotherapy is mainly guided by the local condition of reimbursement, the preferred route of administration by the patient himself and, eventually, by the results of network meta-analyses suggesting a better efficacy of one biotherapy compared to another [1, 2]. The choice may also be influenced by the results of a phase 3b face-to-face trial, which compared vedolizumab and adalimumab in patients with moderate to severe UC [3]. Indeed, the Varsity study has demonstrated that vedolizumab was superior to adalimumab to achieve clinical remission, endoscopic improvement and histologic remission but, when looking at the absolute percentages of clinical remission in both groups, at least 70% and 60% of patients did not achieve improvement at the end of the trial. Moreover, Varsity and other face-to-face trials cannot discriminate patients who are susceptible to respond to one but not to the other biotherapy. Additionally, the efficacy of the second-line biotherapy may be impacted by the first-line biotherapy as it has been suggested for vedolizumab in ulcerative colitis following anti-TNFs loss of response [4]. Thus, there is a huge need of predictors of efficacy of biotherapy in patients with UC.

Vedolizumab binds specifically to α4β7, inhibiting the binding of α4β7-expressing cells to mucosal addressin cell adhesion molecule-1 (MAdCAM-1) and fibronectin. α4β7 is expressed on several types of cells including B cells, eosinophils and natural killer (NK) cells but mainly memory T cells. By binding to the α4β7 integrin, vedolizumab blocks the migration of these cells to the site of inflammation into the gut.

Many studies have explored predictors of biological efficacy focusing on blood immunologic factors, patient’s characteristics and genes related to cytokine [5–8]. At a mucosal level, detection of α4β7-expressing cells in patients with Crohn’s disease (CD) was associated with a response to vedolizumab [9]. In this preliminary study, confocal laser endomicroscopy (CLE) was used to identify, in 5 patients with CD refractory to anti-TNF, *ex vivo* positive staining cells by fluorescein-labelled vedolizumab. Pericryptal α4β7+ cells were identified only in responders [9]. A similar technic of CLE was already used by the same group in a previous study to identify *in vivo* mucosal positive staining cells by fluorescein-labelled adalimumab [9]. The authors have demonstrated that a high number of positive cells into the mucosa of patients with CD was significantly associated with a high rate of response to adalimumab [10].

The aim of our study was to determine the feasibility of *ex vivo* identification of α4β7- and TNF-expressing cells, to predict the clinical, endoscopic and histologic responses to vedolizumab and adalimumab, in biotherapy-naïve UC patients using a new technic of dual laser probe-based CLE system.

## Methods

### Study design

The DETECT study is an open label, prospective, multicenter cohort. Its primary objective was to evaluate the use of dual-band laser probe-based CLE as a tool to predict clinical, endoscopic and histologic responses to vedolizumab or adalimumab in patients with active moderate-to-severe UC naïve of biotherapy. Secondary objectives were to identified blood biomarkers of efficacy of vedolizumab. The study was conducted in 4 sites from 2016 through 2020. The trial protocol was approved by an institutional review board and ethics committee (CPP ouest IV reference: 23/16) on June 2016 and, all the patients provided written informed consent. The protocol was registered under the number NCT02878083 in Clinical-trial.gov and under the number 2016-001130-96 in the EudraCR database.

### Patients

Patients with ulcerative colitis referred or followed-up in 4 French centers were evaluated for eligibility. Inclusion criteria were: adults over 18 years with moderately to severely active ulcerative colitis defined as a total score of 6 to 12 on the Mayo scale and a subscore of at least 2 on the endoscopic component of the Mayo scale [11], with a colonic involvement of at least 15 cm, no response or loss of response or intolerance to conventional treatments, and naïve of biotherapy.

Screening assessment, performed at the Clinical Investigation Centre of the Institute of Digestive diseases (Nantes), included a physical examination, an endoscopy, the total Mayo score, blood tests, blood samples, and colonic biopsies for CLE and histologic analysis.

All included patients received intravenous infusions of vedolizumab 300 mg on day 1 and weeks 2, 6 and 14. Responders at week 22 continued vedolizumab every 8 weeks and non-responders received subcutaneous adalimumab 160 mg at week 22, 80 mg at week 24 and then 40 mg every-other-week.

### Follow-up assessments and definition of efficacy

Trial visits occurred at week 2, 6, 14 and 22 for responders to vedolizumab and through week 30 for non-responders to vedolizumab subsequently treated by adalimumab. A partial Mayo score which consisted of three of the four components of the Mayo scale (stool frequency, rectal bleeding and physician’s global assessment) was calculated at inclusion and at each follow-up visit. The total Mayo score was calculated at inclusion and week 22 for all the patients and at week 30 for the patients treated by adalimumab after vedolizumab.

Clinical remission at week 22 was defined as a total score ≤ 2 and no subscore > 1. Other outcomes were corticosteroid-free clinical remission, clinical response defined by a decrease of the partial Mayo score ≥ 3 points and of 30% decrease from the baseline value with stool frequency score ≤ 1 and rectal bleeding score ≤1, endoscopic improvement defined as a subscore of 0 or 1 on the Mayo endoscopic component, histologic response defined as a Geboes score < 3.1.

### Exploratory measures

Flexible sigmoidoscopy was performed by a trained endoscopist, in non-sedated patients, using a standard colonoscope (EC530, Fujinon), up to 35cm from the anal margin at inclusion and week 22 in all patients. A third sigmoidoscopy was performed at week 30 for the patients no-responders to vedolizumab at week 22 subsequently treated by adalimumab. Eight biopsies were taken in inflamed area using standard biopsy forceps without needle (FB230U; Olympus co; Rungis, France), two for histologic analyses, 3 for ex vivo dual-band CLE 3 for immunofluorescence. Blood samples were collected for FACS analysis and measure of trough concentration (TC) of vedolizumab.

### Antibodies labeling

Adalimumab (Humira 40 mg, Abbvie, 0,8mL solution for injection in pre-filled pen) and Vedolizumab (Entyvio 300 mg, Takeda, powder for concentrate for solution for infusion) were obtained from the Nantes University Hospital Pharmacy.

Before labelling procedure, Adalimumab and Vedolizumab were diluted to 2 mg/mL within 0.05M Na^+^ borate buffer. Antibodies labeling was performed according to APEX™ Antibody Labeling Kits protocol (Invitrogen™ #53027). This kit allowed to covalently attaching Alexa Fluor™ 647 to Adalimumab and FITC™ 488 to Vedolizumab. At the end of the procedure, presence of labelled antibodies was assessed by dot blot. Labelled-antibodies were stored at -80°C in aliquots of 20μg of proteins in PBS buffer with 0.1% NaN_3_.

### Confocal laser endomicroscopy

The dual-band CLE imaging measurement data were recorded with the Cellvizio™ system. FITC-labelled vedolizumab and Alexa fluor-labelled adalimumab were applied on mounted fresh colonic biopsies at a concentration of 2 mg/mL during 1 min. The dual-band CLE system was loaned by Cellvizio Lab. (Mauna Kea Technologies, France). The dual-band CLE images were acquired using the UltraMiniO probe under 488 nm (for FITC) or 660 nm (for Alexa Fluor) laser excitation. At 488 nm excitation wavelength, the emission wavelength range was 502–633 nm. At the excitation wavelength of 660 nm, the emission wavelength range was 673–800 nm. The CLE Probe characteristics were: diameter 2.6 mm; lateral resolution 1.4 μm; working distance 60 μm; maximal field of view 240 μm; frame rate: 8–12 frames per second.

The CLE images were mosaicked to expand the imaging field with a high resolution.

After image acquisition with CellvizioTM system for each wavelength, movies were converted to mp4 and analyzed on the Fiji ImageJ v1.53f51 software using the following macro. A Z projection in maximum intensity was performed in order to superimpose all the images obtained by each biopsy. One acquisition was performed by patient. After thresholding, the rate of patients in whom biopsy staining was detected using CLE, the number of areas ≥ 70 μm^2^ corresponding to the approximative surface of one lymphocyte, the total area in μm^2^ positively stained and the percentages of the whole image stained by both antibodies were determined.

The analysis was blinded to the clinical and endoscopic results.

### FACS analysis

Absolute counts of T cells, B cells, NK cells and monocytes were determined by flow cytometry (BD FacsCanto II cytometer with DIVA software) and Sysmex XS-800i analyzer in responder and non-responder patients at inclusion, before the first infusion of vedolizumab, and at week 22. In the same way, α4β7 expression was analyzed by flow cytometry using a FITC-conjugated vedolizumab antibody in the same cells and in B cell subpopulations i.e. Plasmablasts, Transitional B cells, Switch and non-Switch B cells, Naïve B cells.

Within a maximum of 4h after drawing, EDTA whole blood samples (50μl) were incubated 15min with the following antibodies to analyze T cells, B cells, monocytes and NK cells: CD45-KrOrange, CD16-PE, CD14-PECy5, IgG1-FITC (Beckman Coulter, France) and with CD3-BV421, IgG1-APC, CD56-PE, CD19-PECy7, CD8-APC-H7 (BD Biosciences, France). For B cells subpopulation analysis, the following antibodies were used for staining 100μl of washed blood: CD45-KrOrange, IgG1-FITC (Beckman Coulter) and CD24-PE, CD27 PerCP-Cy5, CD38-APC, IgD-APC-H7, CD19-PECy7 (BD Biosciences). Quantitation of lymphocyte subsets (B cells, T cells, CD4 and CD8 T cells and NK cells) was performed using flow cytometry with BD Trucount™ Tubes (BD Biosciences).

### Vedolizumab trough concentrations

Vedolizumab TC were measured in all patients just before each infusion of vedolizumab. Sera were collected and stored at -20°C until the end of the study. Vedolizumab TC were determined using a quantitative enzyme-linked immunosorbent assay according to the manufacturer (LISA tracker; Theradiag).

### Immunofluorescence staining from UC colon mucosa

Sections obtained from biopsies frozen in tissue-Tek were fixed for 15 min in paraformaldehyde and rehydrated. Mouse IgG1 anti-α4β7 recombinant antibody (clone mAb ACT-1; ref PABL-746-HRP from Creative Biolabs) or isotype (mouse IgG1, DDXCMO1P from Dendritic products) were incubated at room temperature overnight. After washing, the α4β7 signal was amplified and DAPI (Molecular Probes, D1306) was incubated 30 min. Slides were mounted with Vectashield® Vibrance™ Antifade Mounting Medium (Vector Laboratories). Large images were acquired with the Nikon A1RSi confocal resonant microscope using the Nikon denoise.ai algorithm. The images were analyzed with the Qupath software.3. The number of specifically labeled α4β7 cells was expressed as the rate of α4β7 positive cells over the total number of cells present on the section.

### Statistical analysis

Analyses were performed using R Project software (version 3.5.2). For all statistical analyses, α = 0.05 was considered as an acceptable threshold for type I error.

Efficacy was analyzed in the full-analysis set, that is all patients who verified all inclusion criteria and who received at least one dose of vedolizumab. Due to the exploratory nature of the study and, in the absence of existing published data, it was agreed to include 25 patients.

Continuous variables are described using median and first and third quartile [25th-75th] and were tested using unpaired or paired (for evolutions) Wilcoxon or Mann Whitney non-parametric test for continuous variables. Categorical variables are described as raw counts and percentages and were tested by Fisher’s exact test. The association between the number of areas ≥ 70 μm^2^ stained by the FITC-conjugated vedolizumab or the Alexa fluor-conjugated adalimumab detected by CLE at inclusion and the clinical remission, the endoscopic improvement and the histologic response to vedolizumab at week 22 were tested using logistic regression and expressed as Odd ratios [Confidence Interval 95%] and area under the curve (AUC) of receiver operating characteristics (ROC). The “optimal” cutoff was estimated using a maximization of the metric and then harmonized according to the different endpoints.

## Results

### Patients’ characteristics

**Fig 1:**
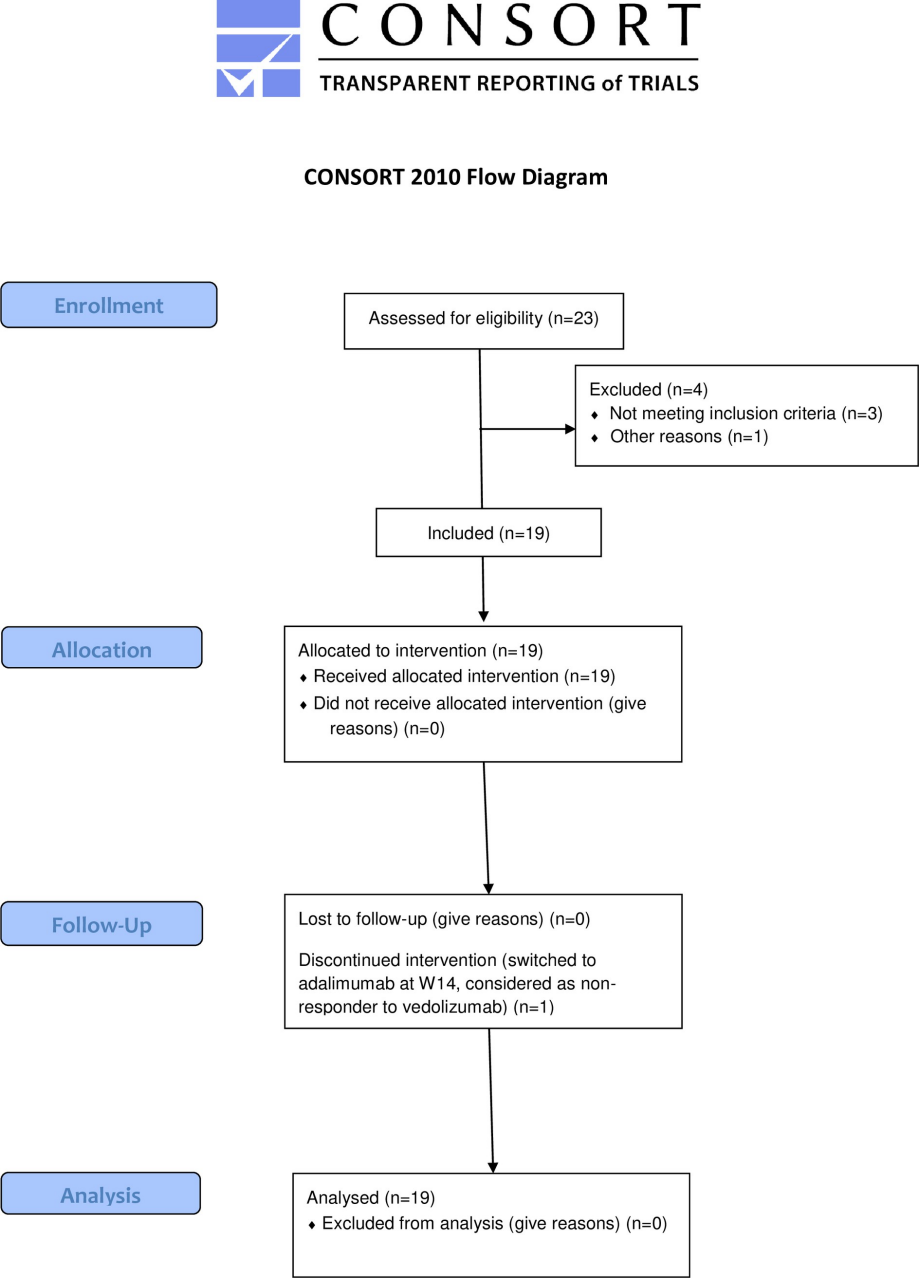
DETECT study flow chart. A total of 23 patients were screened for eligibility and 19 were enrolled in the study and received at least one dose of vedolizumab. Three patients did not meet the inclusion criteria and one patient declined its participation. All patients received vedolizumab at day 0 and week 2, 6 and 14 and were evaluated at week 22. One patient who discontinued vedolizumab between day 0 and week 22 and, who received prematurely adalimumab was considered as a failure of vedolizumab and was analyzed at week 30.

Due to a delay in recruitment compared with objectives, with an initial study extension of 12 months, a total of 23 patients, among the 25 initially anticipated, were screened for eligibility and 19 were enrolled in the study and received at least one dose of vedolizumab between January, 2017 and January, 2020. Last patient was followed up until July, 2020 Three patients did not meet the inclusion criteria and one patient declined its participation. The characteristics of the patients are detailed in Table 1. All patients received vedolizumab at day 0 and week 2, 6 and 14 and were evaluated at week 22. One patient who discontinued vedolizumab between day 0 and week 22 and who received prematurely adalimumab, was considered as a failure of vedolizumab and was analyzed at week 30. The CONSORT flow chart of the study is shown in Fig 1.

**Table 1:** Patient demographic and disease characteristics at baseline.

	Patients (n = 19)
Age-yr, mean (SD)	39.9 (13.3)
Male sex, n (%)	5 (26)
Body weight-Kg, mean (SD)	70.8 (11.9)
Current smokers, n (%)	2 (11)
Duration of ulcerative colitis-yr, mean (SD)	4.2 (4.3)
Extension of ulcerative colitis, n (%)	
E2	12 (63)
E3	7 (37)
Treatments at inclusion, n (%)	
Amino-salicylates	8 (42)
Corticosteroids	6 (32)
Azathioprine	3 (16)
Partial Mayo score at inclusion, mean (SD)	6.0 (1.7)
Total Mayo score at inclusion, mean (SD)	12.1 (3.5)
Endoscopic Mayo score at inclusion, n (%)	
Mayo 2	9 (47)
Mayo 3	10 (53)
Histologic score, med (IQR)	4.3 [3.6–5.2]

### Feasibility of CLE detection after colonic biopsy staining

**Fig 2:**
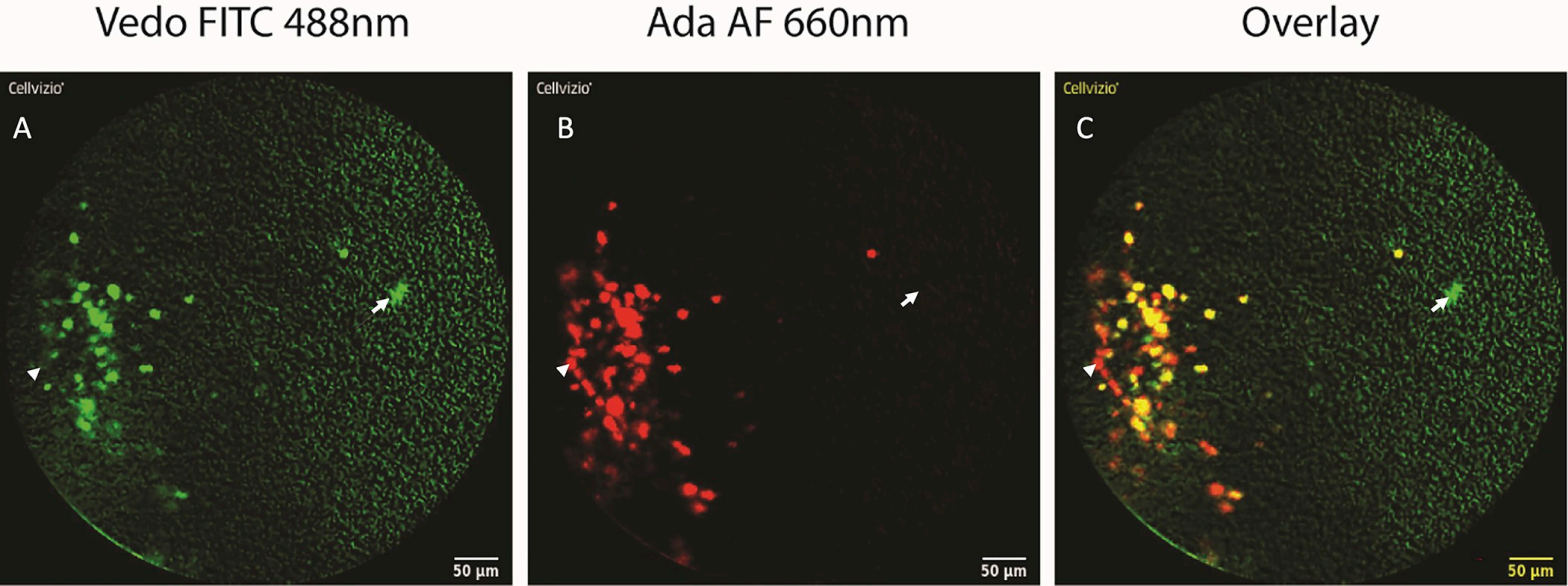
Representative CLE pictures obtained from the same colonic biopsy after staining with vedolizumab-FITC (panel A, green) and adalimumab-Alexa fluor (panel B, red). Arrows identified positive vedolizumab -FITC positive cells and solid arrows, adalimumab-Alexa fluor positive cells in each panel. CLE images were merged (panel C) and showed an overlay of 80%.

Fresh biopsies taken in inflamed area were obtained in 18 out of 19 patients and were used for CLE analysis. In one patient, fresh biopsies were not provided to the lab for unknown reason. After applying FITC-labelled vedolizumab and Alexa fluor-labelled adalimumab, positive staining was detected for both antibodies in 17 (94%) samples and were suitable for analysis (Fig 2). In only one case, the detection of area stained by both antibodies using CLE was impossible. No reason was identified to explain this technical failure. The number of areas ≥ 70 μm^2^ positively stained, the total area in μm^2^ positively stained and the percentages of the whole image stained by both antibodies are detailed in Table 2 (all comparisons by a Wilcoxon signed rank test).

**Table 2:** Comparison of FITC-labelled vedolizumab and Alexa fluor- labelled adalimumab staining detected by CLE in inflamed and non-inflamed mucosa. Results are expressed as median [IQR].

	FITC-labelled vedolizumab	Alexa fluor-labelled adalimumab	Individual differences	p value
	Inflamed mucosa (n = 17)			
Number of areas with positive staining (≥70 μm^2^)	6.2 [3.0–9.8]	7.0 [5.8–11.3]	-1.5 [-2.7; 0.2]	0.070
Total area of positive staining, μm^2^	857.5 [448.3–1694.8]	1511.0 [1056.3–2590.7]	-620.0 [-1128.0; -323.0]	0.004
Percentage of positive staining into the image	0.3 [0.1–0.5]	0.4 [0.3–0.8]	-0.2 [-0.3; -0.1]	1.000
	Non-inflamed mucosa (n = 4)			
Number of areas with positive staining (≥70 μm^2^)	7.2 [3.0–7.8]	9.2 [2.4–13.0]	0.6 [-1.5; 2.0]	0.880
Total area of positive staining, μm^2^	1115.7 [607.3–1385.4]	1621.5 [323.8–1682.0]	-221.0 [-506.0; 225.0]	0.810
Percentage of positive staining into the image	0.3 [0.2–0.5]	0.5 [0.1–0.5]	-0.1 [-0.2; 0.1]	0.810

At inclusion, the median [IQR] number of areas ≥ 70 μm^2^ stained by the FITC-conjugated vedolizumab tended to be lower than the number of areas stained by the Alexa fluor-conjugated adalimumab on the same biopsies: 6.2 [3.0–9.8] versus 7.0 [5.8–11.3] (p = 0.07). The total surface positively stained (median [IQR]) was significantly lower with the FITC-conjugated vedolizumab (857.5 μm^2^ [448.3–1694.8]) than with the Alexa fluor-conjugated adalimumab (1511.0 μm^2^ [1056.3–2590.7]; p = 0.004). CLE images of biopsies stained by the FITC-conjugated vedolizumab and by the Alexa fluor-conjugated adalimumab were merged and showed a median [IQR] overlay of 80% [68–87] (Fig 2).

Fresh biopsies were obtained from non-inflamed mucosa in only four patients. There was no numeric difference between inflamed and non-inflamed mucosa concerning all the parameters tested and for both antibodies (Table 2).

### Efficacy

Clinical remission at week 22 was observed in 58% (11 of 19) of patients and corticosteroids free-clinical remission in 53% (10 of 19) of patients. The median [IQR] total Mayo score was 12.0 [10.0–14.0] at inclusion and decreased to 2.5 [1.0–7.5] at week 22 (individual differences -7.5 [-12; -6], Wilcoxon paired test p<0.001). The median [IQR] partial Mayo score decreased from 6.0 [5.0–7.0] at day 0 to 1.0 [0.0–4.7] at week 22 (individual differences -3.5 [-6; -1]). The difference was already statistically significant from week 2 (p<0.01) and gradually increased until week 22 (p<0.0001). Endoscopic improvement at week 22 was observed in 53% (10 of 19) of patients, of whom 5 patients (45%) had an endoscopic subscore of 0. Histologic response was observed in 53% (10 of 19) of patients. Ten patients were both in clinical and endoscopic remission, one patient was in clinical remission without endoscopic remission.

At week 22, 6 patients received an induction scheme of adalimumab. One additional patient who received adalimumab at week 14 was also evaluated at week 30. Clinical response, clinical remission and endoscopic improvement at week 30 were observed in 57% (4 of 7), 29% (2 of 7), 43% (3 of 7) of patients respectively. None of the patients with an endoscopic subscore of 0 or 1 had a histologic response.

### Analysis of CLE-detected biopsy staining and response to treatment

In terms of clinical response and remission, endoscopic improvement and histologic response, the number of areas ≥ 70 μm^2^ and the total surface in μm^2^ positively stained by the FITC-conjugated vedolizumab, in biopsies obtained before the initiation of the treatment, tended to be numerically higher in responder patients compared to non-responders at week 22 (Table 3). Conversely, there was no significant difference between responders and non-responders to vedolizumab at week 22 in terms of staining using the Alexa fluor-conjugated adalimumab (S1 Table).

**Table 3:** Vedolizumab staining of colonic biopsies detected by CLE at inclusion and response to vedolizumab at week 22. Results are expressed as median [IQR].

	FITC-labelled vedolizumab (n = 17)			
	Number of areas with positive staining (≥70 μm^2^)	p-value	Total area of positive staining, μm^2^	p-value
Clinical response				
Yes	7.7 [3.7–12.2]	0.3	1146.2 [580.0–1888.3]	0.6
No	5.0 [3.1–5.8]		718.2 [405.7–1000.6]	
Clinical remission				
Yes	8.0 [6.7–13.0]	0.1	1435.0 [654.0–1952.0]	0.2
No	4.1 [2.5–7.6]		583.2 [322.9–995.3]	
Endoscopic improvement				
Yes	8.0 [6.7–13.0]	0.1	1435.0 [654.0–1952.8]	0.2
No	4.1 [2.5–5.7]		583.3 [322.9–995.3]	
Histologic response				
Yes	8.0 [6.7–13.0]	0.07	1435.0 [654.0–1952.8]	0.2
No	4.0 [2.3–5.7]		636.8 [386.9–995.3]	

**Fig 3:**
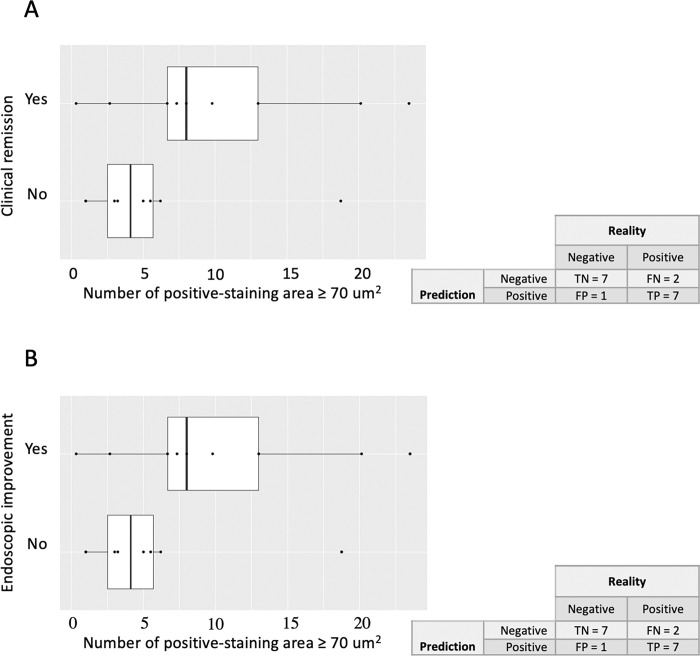
Vedolizumab staining of colonic biopsies detected by CLE at inclusion and response to vedolizumab at week 22. The median of areas ≥ 70 μm^2^ stained by the FITC-conjugated vedolizumab detected by CLE in inflamed colonic biopsies per patient with UC is plotted, with each dot representing one patient. As overlay, a box plot is shown, with the ends of the box representing the first and third quartiles and the middle line the median. Data for clinical remission after vedolizumab at week 22 with the confusion matrix are shown in panel A and endoscopic improvement, defined by a Mayo endoscopic sub-score or 0 or 1, with the confusion matrix in panel B.

The mean (SD) number of areas stained by the FITC-conjugated vedolizumab was 5.5 (5.7) in non-responders versus 10.2 (7.6) in responders which means an Odds Ratio (OR) of 1.13 [CI95: 0.97–1.41] and an area under curve (AUC) of 0.74 (p = 0.2). Similar results were obtained concerning the association of areas stained by the FITC-conjugated vedolizumab at week 0 and histologic response at week 22: OR 1.13 [CI95: 0.97–1.43]; AUC 0.77 (p = 0.2). A threshold value of 6 positive FITC-vedolizumab staining areas detected by CLE before the initiation of the treatment seemed informative to discriminate the responders and non-responders at week 22. For the patients with ≤ 6 positive-staining areas detected by CLE, 6 among 8 (75%) were non-responders. On the contrary, for the patients with > 6 positive-staining areas, 7 among 9 patients (78%) were responders (Fig 3). The results were similar in terms of clinical remission and endoscopic improvement with a sensitivity of 78% and a specificity of 85% (p = 0.05; Fisher’s exact test).

Conversely, there was no signal in favor of the association of positive Alexa fluor-adalimumab staining with clinical or endoscopic evolution of the patients at week 22. For all the parameters tested, the areas under the curves (AUC) of the receiver operating characteristic (ROC) were near 0.5. Similarly, there was no difference between responders and non-responders to adalimumab at week 30 in terms of Alexa-fluor conjugated adalimumab staining at inclusion (S2 Table).

Colonic biopsies were usable in 15 patients, (9 responders) for confocal immunofluorescence staining. We did not observe a significant difference in the rate of α4β7 positive cells between responder and non-responder patients (0.71 vs 0.74%), p = 0.86) (S1 Fig).

### Blood distribution of B and T cells, monocytes and NK cells

At inclusion, there was no difference in absolute counts of T cells, B cells, NK cells and monocytes between responders and non-responders to vedolizumab. In responder patients, the mean absolute count of monocytes decreased significantly between week 0 and 22 from 586 to 398 cells/μl (p = 0.033) and monocytes were also significantly fewer at week 22 in responders compared to non-responders (398 versus 648 cells/μl; p = 0.036) (S2 Fig).

Similarly, concerning the expression of α4β7 in a similar subset of cells, there was no difference between responders and non-responders to vedolizumab before the initiation of the treatment. At week 22, the number of vedolizumab positive B cells decreased significantly in both responders and non-responders: 8.7 versus 0.2 and 9.6 versus 0.1 cells/μl respectively (p = 0.003 and 0.03) (S2 Fig). B cells subpopulations were explored and confirmed the absence of difference between responders and non-responders to vedolizumab before the initiation of the treatment. Mean plasmablasts and switch B cells decreased between inclusion and week 22 in both groups and the difference was only significant in responder patients: 3.6 versus 0.1 cells/μl (S3 Fig).

### Vedolizumab trough concentrations

Serum samples were obtained in 19 patients; 11 patients were in clinical remission at week 22. Vedolizumab TC at week 22 were undetectable in two non-responder patients. The mean concentration of vedolizumab at week 22 was significantly higher in responders compared to non-responders: 22.1 μg/mL versus 18.7 μg/mL, p<0.01. No association was observed between vedolizumab TC at weeks 2, 6 and 14 and response at week 22.

## Discussion

Vedolizumab is a monoclonal antibody targeting the α4β7 heterodimer that was developed to limit lymphocyte trafficking to the intestine. The mechanisms of action of vedolizumab are not fully understood but it was demonstrated that it reduces the adhesion of CD4 and CD8 effector T cells to MAdCAM-1 [12]. Other cell types expressing α4β7 may be targeted by vedolizumab such as B cells, NK cells and CD103+ (subunit of aEb7 integrin) cDCs [13]. Even if other mechanisms of action, not yet identified, could explained in part the efficacy of vedolizumab, it seems that decreasing the gut infiltration by immune cells expressing α4β7 plays a major role.

Because of the mechanism of action of vedolizumab, we explored the potential link between the cellular infiltrate expressing α4β7 and its therapeutic efficacy using an endomicroscopy technique of detection. This was underpinned by the results of a phase II study in which the efficacy of etrolizumab, another blocking monoclonal antibody anti-b7 integrin subunit, was associated with a high expression of aE gene expression into the colonic mucosa of UC patients [14]. The choice of CLE to detect mucosal cells expressing α4β7 was also guided by the demonstration of its feasibility in patients with CD treated by anti-TNF [10]. In this study, it was demonstrated that the efficacy of adalimumab was strongly associated with a high proportion of mucosal cells labelled by adalimumab-FITC. In our study, we have used the Cellvizio dual-band CLE that allowed to simultaneously identify two distinct markers using two different wavelengths of excitation. Thus, it was theoretically possible to identify in a one-time procedure mucosal cells expressing α4β7 and cells expressing transmembrane TNF using labeling with vedolizumab coupled with FITC and adalimumab coupled with Alexa Fluor. We have demonstrated the feasibility of the procedure with more than 90% of biopsies usable for measuring markers expression. The number of positive FITC-vedolizumab areas and the total surface positively labeled were numerically higher for all clinical, endoscopic and histologic criteria in responder patients compared to non-responders but differences did not reach significance. Contrary to what was observed with FITC-vedolizumab, there was no signal in favor of the association of positive Alexa fluor-adalimumab staining with clinical or endoscopic evolution after induction therapy by vedolizumab. We were also unable to reproduce the results published by Atreya R et al., in patients treated by adalimumab after the primary non-response to vedolizumab but that can be explained by the very low number of patients and the different technique of endomicroscopy [10]. Finally, it seems that beyond the threshold of 6 positively marked areas with vedolizumab-FITC, the probability to respond to vedolizumab was high with a sensitivity of 78% and a specificity of 85%, results to be confirmed on a more important cohort of patients.

At the blood level, the induction therapy by vedolizumab induced a decrease of absolute monocytes and B cells expressing vedolizumab, especially plasmablasts and switch-B cells subpopulations, both in responders and non-responders. There was no signal in favor of a specific cell population associated with a response to vedolizumab. Trough concentrations (TC) were also not informative.

The main strength of this study lies in its prospective and multicenter design and blinded evaluation of CLE images. Indeed, all the clinical and endoscopic objectives were pre-specified and evaluated by the physicians. The histologic analyses were performed by a pathologist blinded to the endoscopic and clinical results and the CLE and blood analyses were also performed by a physician or a clinical study engineer blinded to the efficacy criteria.

Another strength of the DETECT study comes from the CLE technology which has the potential to be used during routine endoscopy. The CLE is widely used in clinical practice for the detection of pre-cancerous lesions and has been evaluated in IBD for the evaluation of mucosal healing or the differentiation between UC and CD [15, 16]. If used *in vivo*, the Dual-band Cellvizio technology would enable to performed a “virtual-biopsy” after an infusion of fluorescein and to use in the same time a fluorescent marker to target a protein of interest.

The DETECT study has some limitations. The main limitation comes from the number of patients included in the study and the lack of power to demonstrate a statistically significant association of a positive mucosal staining of FITC-vedolizumab and the efficacy at week 22. However, this study was designed as a pilot one to demonstrate the potential use of CLE to detect positive FITC-vedolizumab staining on biopsies. The absence of published data prevented us from calculating the sufficient number of patients to include in the study to demonstrate a significant association. We decided to include 25 patients as it was done in the study of Atreya et al. [10]. The second limitation comes from the use of two distinct markers i.e. vedolizumab-FITC and adalimumab-Alexa Fluor. Indeed, the Cellvizio technology allows to analyze in details the mucosal morphology after the i.v. administration of fluorescein during endoscopy. Because vedolizumab was coupled to FITC, we were unable to administer fluorescein and to analyze *ex vivo* the mucosa and to localize the positive cells. Confocal immunofluorescence was used to confirm the presence of specific α4β7 positive cells into the mucosa. Cells were identified in responder and non-responder patients as shown in the S1 Fig.

However, the results obtained in this pilot study pave the way for an *in vivo* study using labelled antibodies targeted against α4β7 responding to the good manufacturing practice (GMP) and the current standards of the health authorities with a different wavelength of the fluorescein. The Dual-band Cellvizio might offer the possibility to explore during an endoscopy both the mucosal architecture after the administration of fluorescein and the positive stained cells expressing α4β7.

## Supporting information

S1 Checklist TREND statement checklist. (DOC)

S1 Fig Representative IFI pictures of slides from formalin-fixed, paraffin-embedded (FFPE) sections of UC colon stained with mAbs against α4β7 (red) and DAPI (blue). Original magnification x 200 (A) & (C). Higher magnification of α4β7+ cells in the colon (B).
(TIF)

S2 Fig Absolute counts of T cells (panel A), B cells (panel B) and NK cells (panel D) as determined by flow cytometry in responders (R) and non-responders (NR) to vedolizumab at week 0 (W0) and week 22 (W22). The monocyte blood count (panel C) was measured using the Sysmex XS-800i analyzer. There was no difference of absolute counts of the different cells between responders and non-responders to vedolizumab. In responder patients, the absolute count of monocytes (panel C) decreased significantly between week 0 and 22 and monocytes were also significantly fewer at week 22 in responders compared to non-responders. α4β7 expression was analyzed by flow cytometry using a FITC-conjugated vedolizumab antibody in T cells (panel E), B cells (panel F), NK cells, monocytes (panel G) and NK cells (panel H). There was no difference between responders and non-responders to vedolizumab at week 0 before the initiation of the treatment. At week 22, the number of vedolizumab positive B cells decreased significantly in both responders and non-responders (panel F).
(TIF)

S3 Fig B cell subpopulations analysis for α4β7 expression by flow cytometry using a FITC-conjugated vedolizumab antibody in responders (R) and non-responders (NR) patients at weeks 0 (W0) and 22 (W22). Plasmablasts and switch B cells decreased between inclusion and week 22 in both groups and the difference was only significant in responder patients.
(TIF)

S1 Table Adalimumab staining of colonic biopsies detected by CLE at inclusion and response to vedolizumab at week 22. Results are expressed as median [IQR].
(DOCX)

S2 Table Adalimumab staining of colonic biopsies detected by CLE and response to adalimumab at week 30. Results are expressed as median [IQR].
(DOCX)

S1 Protocol (PDF)
