# Supplementary figures and images for: Confocal laser endomicroscopy as predictive biomarker of clinical and endoscopic efficacy of vedolizumab in ulcerative colitis: The DETECT study
Source: PLoS One. 2024 Apr 2;19(4):e0298313. doi: 10.1371/journal.pone.0298313 (PMC10986992; doi:10.1371/journal.pone.0298313)

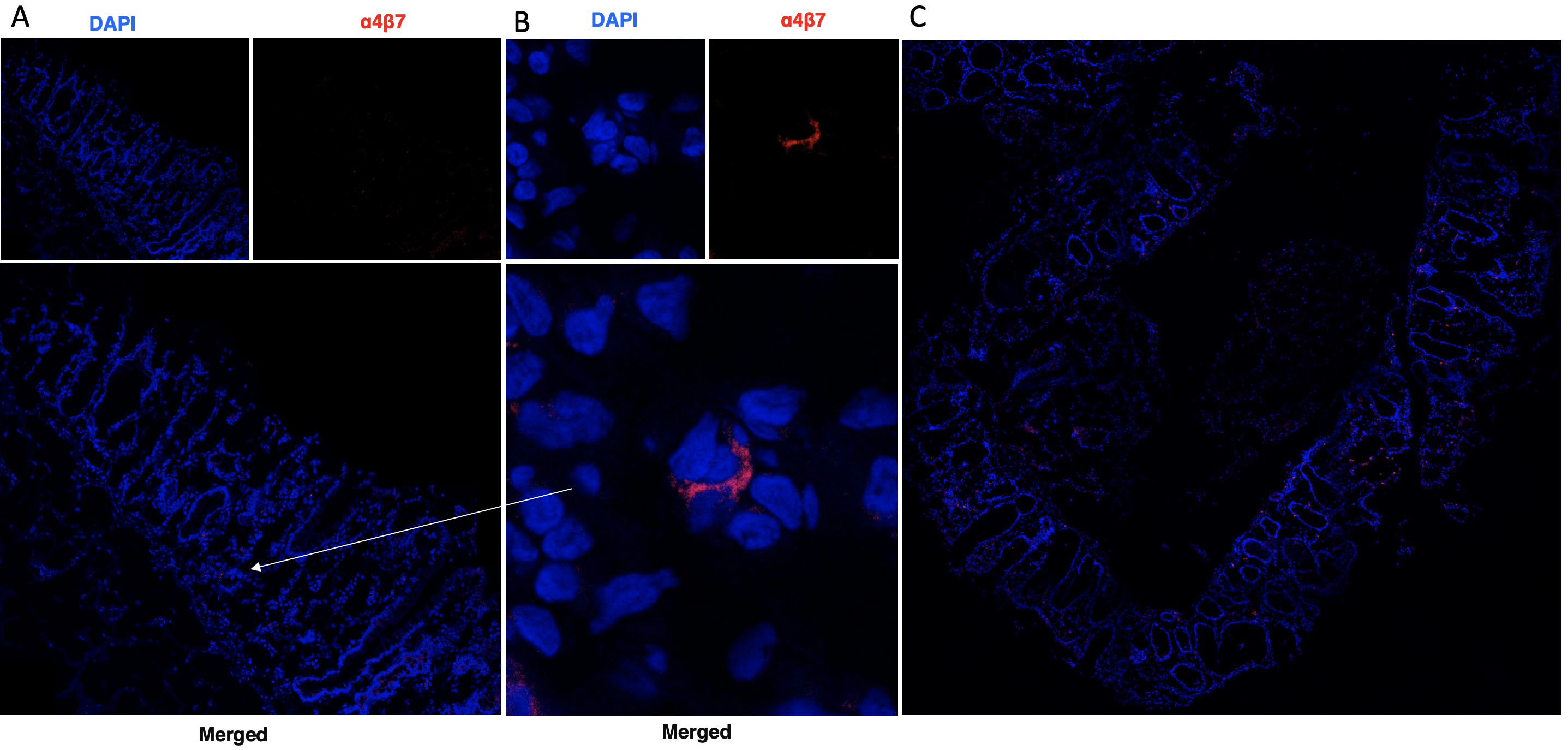

Supplement: S1 Fig — Representative IFI pictures of slides from formalin-fixed, paraffin-embedded (FFPE) sections of UC colon stained with mAbs against α4β7 (red) and DAPI (blue). Original magnification x 200 (A) & (C). Higher magnification of α4β7+ cells in the colon (B). (TIF) [file pone.0298313.s002.tif]

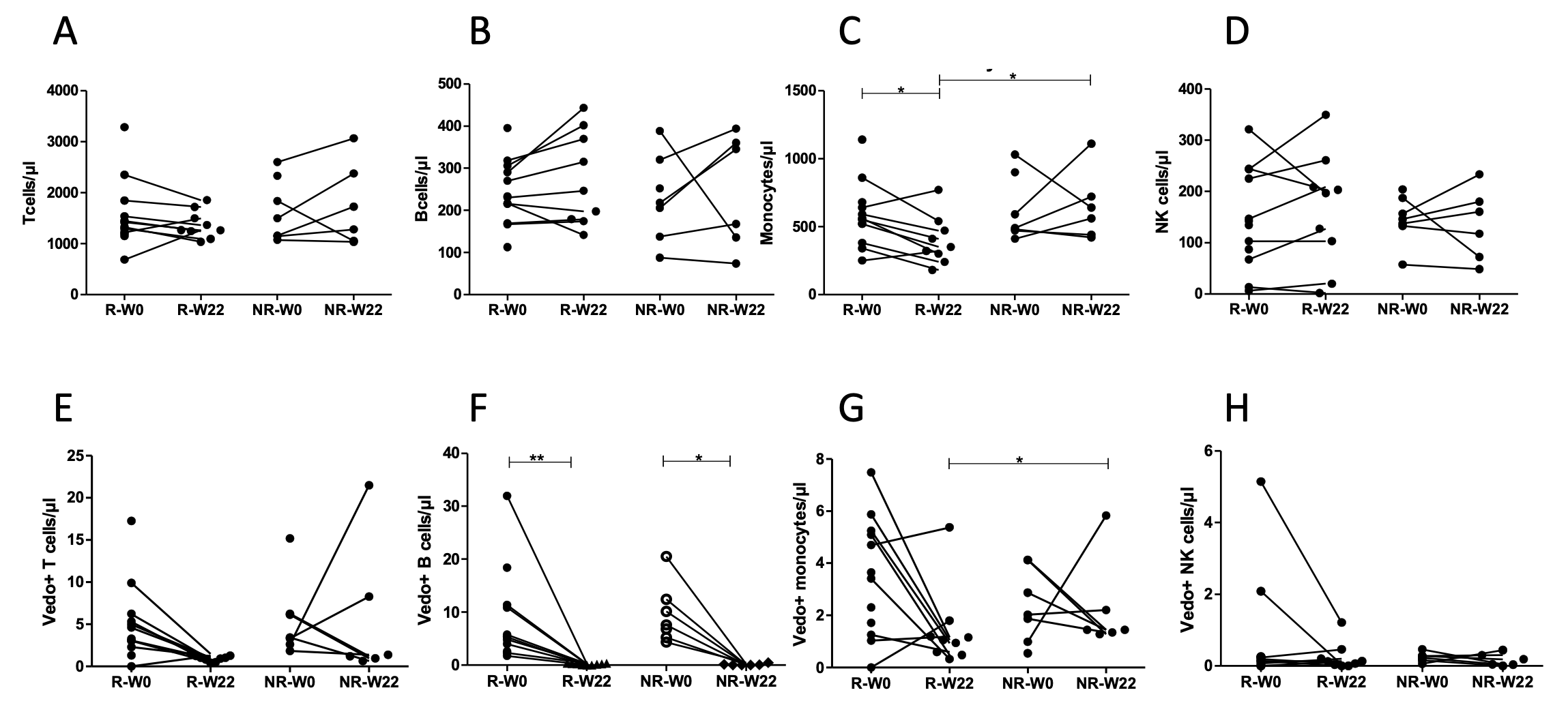

Supplement: S2 Fig — Absolute counts of T cells (panel A), B cells (panel B) and NK cells (panel D) as determined by flow cytometry in responders (R) and non-responders (NR) to vedolizumab at week 0 (W0) and week 22 (W22). The monocyte blood count (panel C) was measured using the Sysmex XS-800i analyzer. There was no difference of absolute counts of the different cells between responders and non-responders to vedolizumab. In responder patients, the absolute count of monocytes (panel C) decreased significantly between week 0 and 22 and monocytes were also significantly fewer at week 22 in responders compared to non-responders. α4β7 expression was analyzed by flow cytometry using a FITC-conjugated vedolizumab antibody in T cells (panel E), B cells (panel F), NK cells, monocytes (panel G) and NK cells (panel H). There was no difference between responders and non-responders to vedolizumab at week 0 before the initiation of the treatment. At week 22, the number of vedolizumab positive B cells decreased significantly in both responders and non-responders (panel F). (TIF) [file pone.0298313.s003.tif]

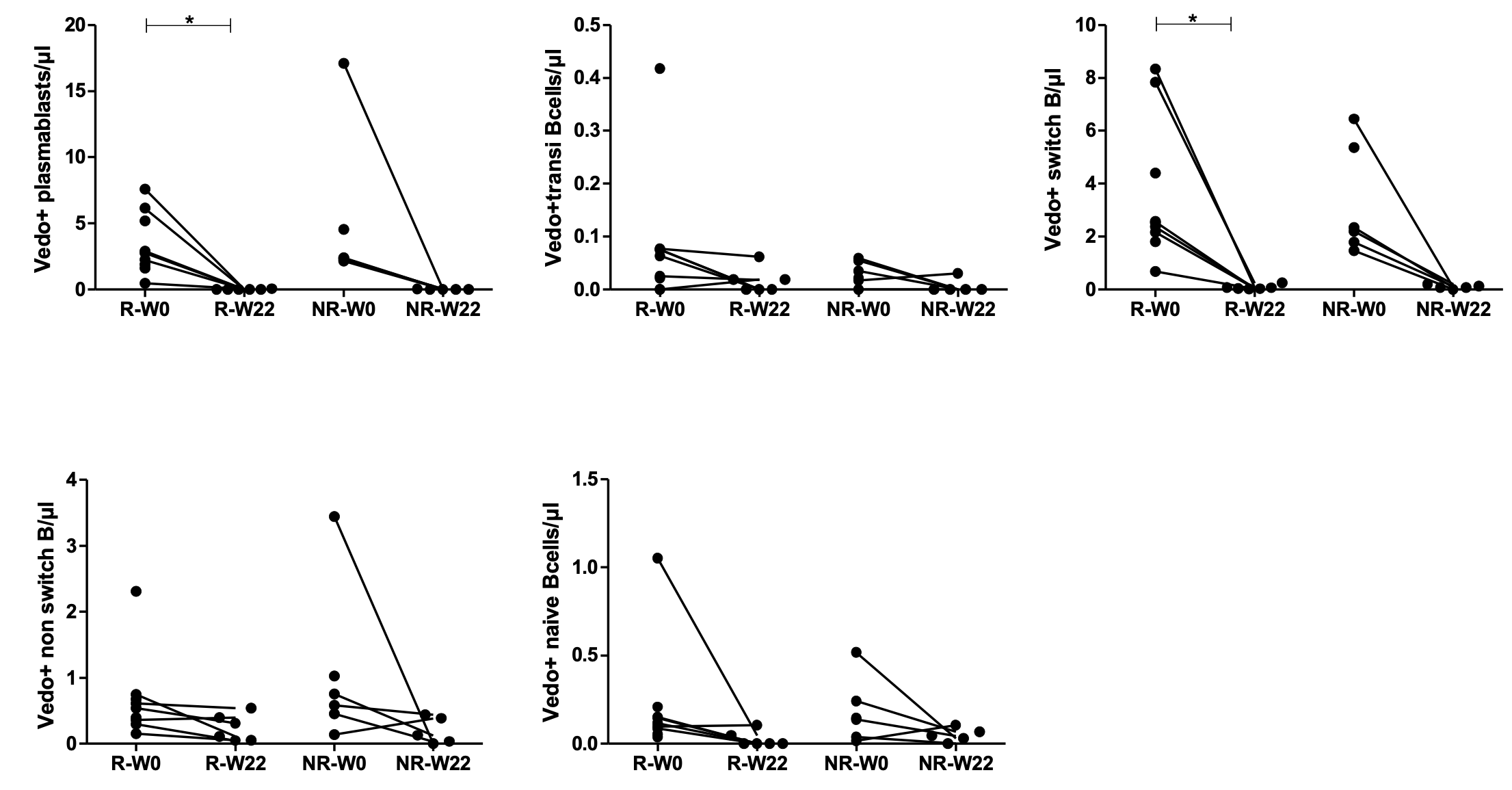

Supplement: S3 Fig — Plasmablasts and switch B cells decreased between inclusion and week 22 in both groups and the difference was only significant in responder patients. (TIF) [file pone.0298313.s004.tif]
