# Supplementary material for: Confocal laser endomicroscopy as predictive biomarker of clinical and endoscopic efficacy of vedolizumab in ulcerative colitis: The DETECT study
Source: PLoS One. 2024 Apr 2;19(4):e0298313. doi: 10.1371/journal.pone.0298313 (PMC10986992; doi:10.1371/journal.pone.0298313)
Supplement: S1 Table — Results are expressed as median [IQR]. (DOCX) [file pone.0298313.s005.docx]

S1 table: Adalimumab staining of colonic biopsies detected by CLE at inclusion and response to Vedolizumab at week 22. Results are expressed as median [IQR]

|  | Alexa fluor-labelled adalimumab (n=17) | | | |
| --- | --- | --- | --- | --- |
|  | Number of areas (≥70 μm^2^) with positive staining | p-value | Total area of positive staining, μm^2^ | p-value |
| Clinical response  Yes  No | 6.5 [3.0 – 11.5]  7.2 [5.8 – 16.0] | 0.5 | 1274.3 [567.7 -2402.1]  1511.0 [1244.7-2428.5] | 0.5 |
| Clinical remission  Yes  No | 6.6 [4.1 – 11.5]  7.1 [5.8 – 7.3] | 0.9 | 1662.7 [650.1-2496.4]  1383.7 [1148.5-2316.4] | 0.9 |
| Endoscopic improvement  Yes  No | 6.6 [4.1 – 11.5]  7.1 [5.8 – 7.4] | 0.9 | 1662.7 [650.1 – 1952.8]  1383.7 [1148.5-2316.4] | 0.9 |
| Histologic response  Yes  No | 6.6 [4.2 – 11.5]  7.1 [5.6 – 7.4] | 1.0 | 1662.6 [700.0 – 2496.4]  1372.0 [1015.6 – 2316.4] | 1.0 |
