# Supplementary material for: Confocal laser endomicroscopy as predictive biomarker of clinical and endoscopic efficacy of vedolizumab in ulcerative colitis: The DETECT study
Source: PLoS One. 2024 Apr 2;19(4):e0298313. doi: 10.1371/journal.pone.0298313 (PMC10986992; doi:10.1371/journal.pone.0298313)
Supplement: S2 Table — Results are expressed as median [IQR]. (DOCX) [file pone.0298313.s006.docx]

S2 table: Adalimumab staining of colonic biopsies detected by CLE and response to Adalimumab at week 30. Results are expressed as median [IQR]

|  | Alexa fluor-labelled adalimumab (N = 7) | | | |
| --- | --- | --- | --- | --- |
|  | Number of areas with positive staining (≥70 μm^2^) | p value | Total area of positive staining, μm^2^ | p value |
| Clinical response  Yes (n=4)  No (n=3) | - 1. [6.7 - 7.4]   7.2 [6.5 - 11.6] | 1 | 1858.0 [1447.0 – 2316.0]  1233.0 [1063.0 – 2400.0] | 0.5 |
| Clinical remission  Yes (n=2)  No (n=5) | 6.6 [6.2; 6.9]  7.2 [7.0; 7.4] | 0.7 | 1730.0 [1493; 1967]  1511.0 [1233; 2652] | 1 |
| Endoscopic improvement  Yes (n=3)  No (n=4) | - 1. [6.6 - 7.4]   2. [6.7 -9.4] | 0.7 | 2204.0 [1730; 2428]  1372.0 [1148; 2025] | 0.5 |
| Histologic response  Yes (n=0)  No (n=7) | 7.2 [6.4 - 7.4] | NA^#^ | 1511.0 [1244.0 – 2428.0] | NA^#^ |

^#^ Not applicable; none of the patients had histologic response at week 30
