# Supplementary material for: Confocal laser endomicroscopy as predictive biomarker of clinical and endoscopic efficacy of vedolizumab in ulcerative colitis: The DETECT study
Source: PLoS One. 2024 Apr 2;19(4):e0298313. doi: 10.1371/journal.pone.0298313 (PMC10986992; doi:10.1371/journal.pone.0298313)
Supplement: S1 Protocol — (PDF) [file pone.0298313.s007.pdf]

[redacted]

DETECT

Eudract : n° 2016-001130-96

Ref : RC15_0457

Ref CPP : 23/16

"Development of an efficacy biomarker for vedolizumab (Entyvio®) in ulcerative colitis".

Coordinating Investigator :

Prof. Arnaud BOURREILLE
Institute of Digestive System Diseases (IMAD)
Nantes University Hospital - Hôtel Dieu
1 place Alexis Ricordeau
44093 Nantes cedex 01
Tel.: 02 40 08 31 52
arnaud.bourreille@chu-nantes.fr

Methodologist :

Prof. Véronique SEBILLE
Nantes University Hospital
INSERM EA4275
5 allée de l'ile Gloriette
44093 Nantes cedex 01
Tel.: 02 40 08 74 43
veronique.sebille@univ-nantes.fr

Promoter :

[redacted]

Nantes University Hospital
Medical Affairs Department
and Research

5, allée de l'île Gloriette
44 093 Nantes cedex 01 (FRANCE)
Tel: 02 53 48 28 35
Fax : 02 53 48 28 36

SIGNATURE PAGE

PROMOTER'S SIGNATURE

The sponsor undertakes to carry out this study in accordance with all the legislative and regulatory provisions to which the research may be subject and in accordance with the protocol.		
**Name and position of signing representative : For the promoter and by delegation of the Chief Executive Officer, the Director of Medical Affairs and Research**	**Date :**	**Signature :**

SIGNATURE OF INVESTIGATOR S

I have read all the pages of the protocol for the clinical trial sponsored by Nantes University Hospital. I confirm that it contains all the information necessary for the conduct of the trial. I undertake to carry out the trial in compliance with the protocol and the terms and conditions defined therein. I undertake to carry out the trial in compliance with :

- the principles of the Helsinki Declaration,
- international (ICH) and French rules and recommendations of good clinical practice (rules of good clinical practice for biomedical research involving medicinal products for human use - decisions of 24 November 2006)
- national legislation and regulations relating to clinical trials,
- compliance with the current European Directive on clinical trials

I also undertake to ensure that the investigators and other qualified members of my team have access to copies of this protocol and documents relating to the conduct of the trial to enable them to work in compliance with the provisions set out in these documents.

**Coordinating investigator**	**Name :**<br><br>Professor Arnaud BOURREILLE<br>Institute of Digestive System Diseases (IMAD)<br>Nantes University Hospital - Hôtel Dieu<br>1 place Alexis Ricordeau<br>44093 Nantes cedex 01<br>Tel.: 02 40 08 31 52<br>arnaud.bourreille@chu-nantes.fr	**Date :**	**Signature :**
**Principal investigator**	**Name and establishment :**	**Date :**	**Signature :**

LIST OF ABBREVIATIONS

ANSM	French National Agency for the Safety of Medicines and Health Products
AMM	Marketing Authorisation
ARC	Clinical Research Associate (monitor)
BPC	Good Clinical Practice
CIC	Clinical Investigation Centre
PPC	Individual Protection Committee
CNIL	Commission Nationale de l'Informatique et des Libertés (French Data Protection Authority)
CRF	Case Report Form (observation booklet)
DCI	International non-proprietary name
eCRF	Electronic Case Report Form (cahier d'observation électronique)
EvIG	Serious Adverse Event
EIG	Serious Adverse Effect
EIGI	Unexpected Serious Adverse Effect
FITC	Fluorescein IsoThioCyanate
GMP	Good Manufacturing Practice
IMAD	Institute of Digestive Diseases
INRA	French National Institute for Agronomic Research
INSERM	French National Institute for Health and Medical Research
ITT	Intention To Treat
IBD	Chronic Inflammatory Bowel Diseases
MC	Crohn's disease
MR	Reference Methodology
PHRC	Hospital Clinical Research Programme
RCH	Haemorrhagic RectoColitis
CPR	Summary of Product Characteristics
SUSAR	Suspected Unexpected Serious Adverse Reaction
TEC	Clinical Study Technician
UMR	Mobile Research Unit
5- ASA	5- AminoSAlycilates

TABLE OF CONTENTS

SIGNATURE PAGE 2

LIST OF ABBREVIATIONS 4

TABLE OF CONTENTS 5

INTRODUCTION 9

1. JUSTIFICATION FOR THE STUDY 10

1.1. POSITIONING OF THE RESEARCH 10

1.2. BENEFITS AND RISKS FOR RESEARCH SUBJECTS 14

1.2.1. Benefits 14

1.2.2. Risks 14

1.2.3. Benefit/risk balance 16

1.3. DESCRIPTION AND JUSTIFICATION OF THE THERAPEUTIC REGIMEN 16

2. OBJECTIVES AND ASSESSMENT CRITERIA 17

2.1. OBJECTIVE AND PRIMARY ENDPOINT 17

2.1.1. Main objective 17

2.1.2. Primary endpoint 17

2.2. OBJECTIVES AND SECONDARY EVALUATION CRITERIA 17

2.2.1. Secondary objective(s) 17

2.2.2. Secondary evaluation criterion(s) 18

2.3. OBJECTIVE AND EVALUATION CRITERIA FOR ANCILLARY STUDIES 19

3. RESEARCH DESIGN 20

3.1. GENERAL RESEARCH METHODOLOGY 20

3.2. DIAGRAM OF THE STUDY 20

4. STUDY POPULATION 21

4.1. DESCRIPTION OF THE POPULATION 21

4.2. INCLUSION CRITERIA 21

4.3. NON-INCLUSION CRITERIA 22

5. TREATMENTS USED DURING THE STUDY 23

5.1. DESCRIPTION OF TREATMENTS REQUIRED AND METHODS OF ADMINISTRATION 23

5.1.1. Investigational medicinal product(s)/comparator 23

5.1.2. Other drugs in the protocol 24

5.2. AUTHORISED AND PROHIBITED MEDICINES AND TREATMENTS 25
5.2.1. Authorised treatments 25
5.2.2. Unauthorised treatments 25
5.2.3. Emergency treatment 25
5.3. METHODS FOR MONITORING COMPLIANCE WITH TREATMENT 25
5.4. INVESTIGATIONAL MEDICINAL PRODUCT CIRCUIT 26
5.4.1. General circuit 26
5.4.2. Storage conditions for investigational medicinal products 26

6. CONDUCT OF THE STUDY 27

6.1. RESEARCH AND ANALYSIS TECHNIQUES 27
6.1.1. Detailed description of efficacy assessment parameters 27
6.1.2. Description of techniques and analyses 28
6.2. STUDY TIMETABLE 30
6.3. IDENTIFICATION OF ALL SOURCE DATA NOT CONTAINED IN THE MEDICAL RECORD 35
6.4. RULES FOR TERMINATING A PERSON'S PARTICIPATION 35
6.4.1. Criteria for premature termination of a person's participation in research 35
6.4.2. Procedures for premature termination of a person's participation in research 35
6.4.3. Criteria for stopping part or all of the research (excluding biostatistical considerations) ... 36
6.5. PATIENT MANAGEMENT AT THE END OF THE RESEARCH STUDY 36
6.6. FINAL REPORT ON THE STUDY 37

7. DATA MANAGEMENT AND STATISTICS 38

7.1. COLLECTION AND PROCESSING OF STUDY DATA 38
7.1.1. Data collection 38
7.1.2. Data coding 38
7.1.3. Data processing 39
7.2. STATISTICS 39
7.2.1. Description of planned statistical methods, including timetable for planned interim analyses 39
7.2.2. Statistical justification of the number of inclusions 40
7.2.3. Predicted level of statistical significance 40
7.2.4. Statistical criteria for discontinuing research 41
7.2.5. Method for taking into account missing, unused or invalid data 41
7.2.6. Managing changes to the initial strategy analysis plan 41
7.2.7. Choosing the people to be included in the analyses 41

8. PHARMACOVIGILANCE AND MANAGEMENT OF ADVERSE EVENTS 42

8.1. DEFINITIONS 42
8.2. SAFETY ASSESSMENT PARAMETERS 43
8.2.1. Specific assessment criteria relating to safety..... *43*
8.2.2. Methods and timetable for measuring, collecting and analysing safety assessment parameters 43
REPORTS WILL BE ANALYSED BY THE PHARMACOVIGILANCE DEPARTMENT, WHICH WILL SUMMARISE THEM. 44
8.3. LIST OF EXPECTED AES 44
8.4. MANAGEMENT OF UNDESIRABLE EVENTS 45
8.4.1. Notification of SAEs/EvIGs *45*
8.4.2. Independent Supervisory Committee *45*
8.5. ARRANGEMENTS AND DURATION OF FOLLOW-UP FOR PEOPLE FOLLOWING THE OCCURRENCE OF UNDESIRABLE EVENTS 45

9. ADMINISTRATIVE AND REGULATORY ASPECTS 46

9.1. RIGHT OF ACCESS TO SOURCE DATA AND DOCUMENTS 46
9.2. MONITORING THE TRIAL 46
9.3. INSPECTION / AUDIT 47
9.4. ETHICAL CONSIDERATIONS 47
9.4.1. Written informed consent *47*
9.4.2. Procedures for obtaining consent in an emergency (if applicable) *47*
9.4.3. Individual Protection Committee *47*
9.5. AMENDMENTS TO THE PROTOCOL 48
9.6. DECLARATION TO THE COMPETENT AUTHORITIES 48
9.7. REGISTER OF PERSONS INVOLVED IN BIOMEDICAL RESEARCH 48
9.8. FINANCING AND INSURANCE 48
9.9. RULES RELATING TO PUBLICATION 48
9.10. FATE OF BIOLOGICAL SAMPLES 49
9.11. ARCHIVING SOURCE DATA 49

LIST OF APPENDICES 0

APPENDIX 1: LIST OF INVESTIGATORS 1

APPENDIX 2: SUMMARY OF THE PROTOCOL 1

APPENDIX 3: BIBLIOGRAPHICAL REFERENCES 1

APPENDIX 5: *GEBOES SCORE* 2

APPENDIX 6: *PATIENT INFORMATION LETTER*..... ERREUR ! SIGNET NON DEFINI.

APPENDIX 7: PATIENT CONSENT FORM....................................ERREUR ! SIGNET NON DEFINI.

APPENDIX 8: RCP VEDOLIZUMAB..ERREUR ! SIGNET NON DEFINI.

INTRODUCTION

Just under half of patients with haemorrhagic rectocolitis require treatment with immunosuppressants or biotherapy because conventional treatments have failed. Two classes of biotherapy are available: anti-TNF-alpha (adalimumab, infliximab, golimumab) and anti-integrin (vedolizumab). The former act in part through their ability to bind transmembrane TNF expressed by intestinal immune cells, while the latter bind integrin □4□7 expressed on the surface of lymphocytes entering the digestive tract. Their efficacy is comparable, with approximately 30 to 40% of patients achieving remission.

The choice of which drug to use first is not based on any scientific data, and it is likely that many patients will respond to only one of the two therapeutic classes.

There is a need to develop biomarkers of efficacy to help select the compound most likely to induce remission.

Recently, a biomarker for the efficacy of adalimumab in Crohn's disease was developed, based on the endomicroscopic detection of adalimumab-binding cells coupled to a fluorescent probe (FITC). The accuracy of the test was close to 90%.

The development of new endomicroscopy probes coupled to two laser sources with two different wavelengths makes it possible to detect, at the same time and during endoscopy, cells binding an anti-TNF coupled to FITC and an anti-integrin coupled to another fluorescent probe. The aims of our project are 1) to demonstrate the feasibility of a test for detecting cells binding vedolizumab-FITC and cells binding adalimumab-Alexa fluor 647 using the cellvizio dualband device (Maunakea technologies) and 2) to assess the association between the number of cells labelled with each of the fluorescent antibodies and the clinical, endoscopic and histological remission and response rates.

1. JUSTIFICATION FOR THE STUDY

1.1. RESEARCH POSITIONING

UC is a disease of young adults between the ages of 30 and 40. The incidence in children is rising in Europe and France, with the overall incidence of IBD doubling between 1987 and 2003, from 3.9 to 7.0 per 100,000 population, of which 52% is UC.
The incidence of UC in France is approximately 6 new cases per 100,000 inhabitants. The European prevalence of UC varies from 21.4 to 294 cases per 100,000 inhabitants, corresponding to around 1 million patients in Europe.
The disease develops chronically, with no possibility of cure. Typically, each symptomatic attack is interspersed with a remission phase of varying duration. Ten percent of patients have an active chronic form that is resistant to the various treatments; the risk of colectomy is around 10% at 10 years, and has fallen since the more widespread use of immunosuppressants (IS) and anti-TNF-α drugs[1] .
In contrast to Crohn's disease (CD), 5-aminosalicylic acid (5-ASA) is remarkably effective in the treatment of mild to moderate UC. Approximately 50% of patients achieve remission after induction therapy with 5-ASA[2] and continued treatment maintains 60% of initial responders in remission[3] . In the event of failure or corticosteroid dependence, patients are treated with IS (azathioprine) or biotherapies including three anti-TNF-α drugs (infliximab, adalimumab, golimumab) and an anti-integrin α4β7 antibody (vedolizumab). Overall, anti-TNF-α agents enable remission to be achieved in 20% to 40% of patients treated for moderate to severe UC resistant to standard treatments, with progressive escape over time in around 40% of patients. Only infliximab has demonstrated efficacy in particular forms of severe acute colitis, and its efficacy is comparable to that of ciclosporin in the short term[4] . Their mechanisms of action are incompletely understood, but they share the ability to bind soluble TNF and membrane TNF expressed on the surface of several cell types involved in the immune response. The binding of anti-TNF to membrane TNF induces apoptotic death of activated lymphocytes, which explains a large proportion of their side effects.
Since 2014, vedolizumab has been granted marketing authorisation for the treatment of moderate to severe UC in primary or secondary failure or intolerance to conventional or anti-TNF-α therapies. In the pivotal trial evaluating vedolizumab in the induction and maintenance treatment of moderate to severe UC, 41.8% of patients treated were in remission at 52 weeks, compared with 15.9% of patients treated with placebo[5] . In the same study, subgroup analysis

showed that vedolizumab was significantly more effective than placebo in patients previously treated with anti-TNF-α.

Vedolizumab is an IgG1 monoclonal antibody that specifically blocks an α4β7 integrin expressed on the surface of T and B lymphocytes and prevents its interaction with its ligand, the MadCam-1 addressin expressed on the surface of gastrointestinal endothelial cells. It should be noted that MadCam-1 expression is increased by several pro-inflammatory cytokines overexpressed during chronic inflammatory bowel disease (IBD), such as Interleukin-1 and TNF.

At present, four antibodies are available for use in the same indication in patients with moderate to severe UC who are resistant or intolerant to, or have lost their response to, conventional treatment. Vedolizumab has obtained authorisation for use comparable to anti-TNFs as first-line biotherapy in Finland, Denmark, Germany, Norway, the Netherlands, the UK, the US and Canada. No study has evaluated the best strategy for sequential use of these treatments. It is likely, but not proven, that patients may benefit from the first-line use of either an anti-TNF-α or vedolizumab. Given that the results of each of these molecules do not allow certain and rapid remission to be achieved in all cases, there is an opportunity to identify and develop efficacy biomarkers for the different biotherapies available.

An "ideal" biomarker should be able to be obtained non-invasively or minimally invasively, with immediate results and moderate cost. UC is a particular situation in which the use of a predictive serum efficacy test would not prevent endoscopic exploration of the digestive tract; in fact, therapeutic objectives have changed since the use of biotherapies and endoscopic healing has become a major objective in patient management. The ease with which it can be performed, combined with the fact that recto-sigmoidoscopy has zero morbidity and the disease is located at a low level, mean that this examination is performed almost systematically in patients before treatment is initiated and during follow-up to assess the therapeutic response. Combining endoscopic assessment with a biomarker of therapeutic efficacy would provide physicians with a single examination, endoscopic confirmation of the indication for biotherapy, assessment of the severity of the disease and guidance on the best treatment to use.

Recently, the relevance of a biomarker that can be used endoscopically was demonstrated in patients with CD prior to initiation of anti-TNF-α treatment[6] . Humira® (adalimumab), like other anti-TNFs, acts in part through its ability to bind to membrane TNF expressed on the surface of immune cells in the intestinal mucosa. In this study, humira® was combined with a fluorescent probe, i.e. FITC, and applied to the intestinal mucosa. Firstly, the authors applied fluorescent adalimumab ex vivo to healthy and inflammatory colonic biopsies taken during standard colonoscopy. Fluorescence was analysed using a confocal endomicroscopy technique (optiscan®). This technique makes it possible to observe the intestinal mucosa at

subcellular magnification and to analyse the colonic crypts, intestinal microvascularisation and cellular infiltrate of the lamina propria during standard endoscopy. Using this technique, the investigators demonstrated: 1) that adalimumab - FITC binds specifically to the membrane TNF of immune cells in the intestinal mucosa, 2) that endomicroscopy enables immune cells expressing membrane TNF on their surface to be visualised and counted, 3) that the proportion of cells expressing membrane TNF varies from one patient to another, 4) the identification of two groups of patients on the basis of the number of positive cells per field of examination.
In the second part, the coupling of adalimumab to FITC was carried out in compliance with industrial standards (Good Manufacturing Practice - GMP) allowing their use in humans in vivo. During standard endoscopy, FITC-coupled antibodies were sprayed onto the mucosa using a catheter spray and the mucosa was examined by confocal endomicroscopy at the same time. As with the ex vivo study, two groups of patients were identified; patients in the first group had a high number (>20/field) of positive cells and patients in the second group had a low number of positive cells. The authors demonstrated that patients with a significant infiltrate of labelled cells had a remission rate of more than 90% after initiation of Humira®, whereas the remission rate in patients with a low infiltrate of labelled cells was less than 15%. The sensitivity, specificity, positive predictive value and negative predictive value of the number of labelled cells for predicting therapeutic response were 85%, 92%, 92% and 85% respectively[6] .
It is possible that this technique could be applied to antibodies directed against integrins expressed on the surface of immune cells and found in the intestinal mucosa. Indeed, there are arguments in favour of an impact of integrin expression in the digestive mucosa. Etrolizumab is an anti-β7 monoclonal antibody that binds to α4β7 and αEβ7 integrins and blocks the homing of CD4+ and CD8+ T lymphocytes and B lymphocytes expressing α4β7. In addition to the efficacy of etrolizumab in UC patients, it has been shown that patients with strong expression of αE detected by molecular biology and immunofluorescence in their mucosa had a significantly higher response rate than other patients[7] .
The main objective of our project is to demonstrate the feasibility of a biomarker for the efficacy of adalimumab or vedolizumab biotherapies in UC by coupling vedolizumab to a fluorescent component, FITC, and adalimumab to Alexa fluor 647. The fluorescence of each of the markers can be analysed and quantified in one step by confocal endomicroscopy (Cellvizio®) using a probe that allows laser excitation at two distinct frequencies (488 and 660 nm). The secondary objectives are to 1) verify the specificity of binding of each of the markers to immune cells expressing α4β7 and membrane TNF in the intestinal mucosa of UC patients, 2) to assess the number of cells positive for each of the markers in UC patients prior to treatment with biotherapy, 3) to assess the clinical and endoscopic remission and response rates as a function of the number of cells expressing α4β7 and/or membrane TNF and visualised by confocal endomicroscopy.

The Institut des Maladies de l'Appareil Digestif (IMAD), which brings together the hepato-gastroenterology and digestive surgery departments and the three research units: UMR INSERM U913, CIC INSERM 1114 and UMR INRA PHAN, has the technical facilities and skills needed to bring this project to fruition:

- The immunophenotypic analysis of mucosal cells labelled with fluorescent antibodies will be carried out by the UMR Inserm U1064 team.
- The manufacture of the anti-$\alpha 4\beta 7$ antibody coupled to FITC and adalimumab coupled to Alexa fluor 647 will be carried out within UMR INSERM 913.
- The endoscopy sector is equipped with confocal endomicroscopy (Cellvizio® Mauna Kea technologies) for ex vivo and in vivo analysis of the fluorescence of the various intestinal mucosal microscopic structures.
- Conventional histological analysis of the biopsies will be carried out in the anatomopathology laboratory at Nantes University Hospital.
- Monitoring and organisation will be provided by IMAD's Clinical Investigation Centre (INSERM CIC 1114).

1.2. BENEFITS AND RISKS FOR RESEARCH SUBJECTS

1.2.1. Benefits

1.2.1.1. Individual profit

There is no individual benefit for the person taking part in the Research.

Demonstrating the feasibility and value of an efficacy biomarker would ultimately make it possible to avoid using an ineffective treatment with potential side-effects for 14 weeks in patients with an active disease, before switching to another drug.

1.2.1.2. Group profit

The benefits of this research are collective. Ultimately, the use of biomarkers should make it possible to predict the efficacy of vedolizumab and thus improve the management of people suffering from UC.
Furthermore, predicting the efficacy of biotherapies would make it possible to target the most appropriate treatment for each patient, and would lead to savings in healthcare costs by avoiding the use of expensive and ineffective treatments.

1.2.2. Risks

1.2.2.1. Individual risk

➢ Physical risks and constraints

Participation in the study requires recto-sigmoidoscopy. This type of examination is performed in routine practice. Confocal endomicroscopy performed at inclusion and at W22 requires recto-sigmoidoscopy to be performed at the Nantes centre, where the cellvizio dualband equipment will be located. Transport will be paid for as part of the protocol. During these examinations, three additional biopsies will be taken to perform the *ex vivo procedure*.
There are also plans to :

-take a 10 mL blood tube at baseline and at W22 to count the number of circulating cells binding vedolizumab and adalimumab
and a 10 mL blood tube before each vedolizumab infusion to quantify residual vedolizumab concentrations (W0, W2, W6, W14), at W22 and at S30 if applicable for patients who have received adalimumab (maximum 6 sampling times per patient)

- Disease-related risks

Inclusion in the protocol does not alter the risks associated with the disease. Treatment will be initiated in accordance with standard recommendations, monitoring will be identical to current practice, and response to treatment will be assessed at W22. Each investigator is free to interrupt the study in the event of a worsening of the disease if he or she considers the patient's condition incompatible with continuing the study.
The risks usually encountered are the continuation or worsening of symptoms: diarrhoea, rectal bleeding and abdominal pain. The potential adverse effects of the treatment (vedolizumab / adalimumab) will not be altered by the study.

- Risks associated with vedolizumab and adalimumab

The drugs used will be within the scope of their MA. The expected AEs are listed in the summaries of product characteristics (SPCs) for the treatments. Both products increase the risk of infections, which are generally benign.
A full list of ADRs can be found in the Pharmacovigilance section.

- Psychological risks and constraints

No risk identified

- Socio-economic risks

Carrying out recto-sigmoidoscopy in Nantes implies a significantly longer treatment period, taking into account transport times for patients included in the other investigating centres. The cost of these procedures is borne by the sponsor.

1.2.2.2. Collective risk

No risk identified

1.2.3. Benefit/risk balance

The identification of a biomarker during a non-invasive endoscopic procedure and the identification of the most effective molecule makes it possible to 1) avoid the use of an ineffective treatment with its adverse effects, 2) shorten the active phase of the disease, 3) shorten the period of inactivity for patients and 4) eliminate the direct costs associated with the use of an ineffective biotherapy. There are no risks involved in carrying out the procedure *ex vivo*.

1.3. DESCRIPTION AND JUSTIFICATION OF THE TREATMENT PLAN

This is an exploratory, prospective, multicentre, uncontrolled study involving uncertainty about the number of patients to be included. No data have been published on cell labelling with vedolizumab-FITC. No data have been published concerning double labelling with vedolizumab-FITC and adalimumab-Alexa fluor 647. No data has been published on the analysis of biopsies in contact with these two antibodies by Cellvizio®. The number of patients (25) was assessed to allow statistical analysis of the results, taking into account possible recruitment over the duration of the study in the investigating centres. The data from the study published by Atreya et al.[6] in 20 patients confirms that the primary objective can be met.

The bibliographical references can be found in the appendix to the document.

2. OBJECTIVES AND ASSESSMENT CRITERIA

2.1. OBJECTIVE AND PRIMARY ENDPOINT

2.1.1. Main objective

To demonstrate the feasibility of *ex vivo* labelling of intestinal immune cells using a combination of two markers : vedolizumab-FITC and adalimumab-Alexa fluor 647 quantifiable at the same time and on the same sample by confocal endomicroscopy using a Cellvizio® probe with two wavelengths (488 and 660 nm) on intestinal biopsies from patients with moderate to severe active UC, to develop a biomarker for the efficacy of vedolizumab.

2.1.2. Primary endpoint

Number of fluorescent cells per field of examination in Cellvizio® for each antibody: vedolizumab coupled to FITC and adalimumab coupled to Alexa fluor 647 at W0 for all patients

2.2. OBJECTIVES AND SECONDARY EVALUATION CRITERIA

2.2.1. Secondary objective(s)

- To quantify, ex vivo, the number of FITC-coupled vedolizumab-labelled cells in the intestinal mucosa of patients with moderate to severe UC associated with clinical remission at **W22*** after initiation of vedolizumab treatment.

- To assess the association between the number of cells labelled with fluorescent vedolizumab and the percentage of ~~clinical remission,~~ clinical response, endoscopic remission and histological remission at **W22*** after initiation of vedolizumab treatment.
- Compare the number of immune cells positive for each of the fluorescent antibodies in the intestinal mucosa of UC patients.
- Assess the number of cells doubly labelled with the two fluorescent antibodies

- To assess the association between the adalimumab biomarker coupled to Alexa fluor 647 quantified at baseline and at **W22*** and the rate of clinical response, clinical remission, endoscopic remission and histological remission to adalimumab in second-line treatment after failure of vedolizumab.
- Immunofluorescence determination of the cellular phenotype of cells labelled with vedolizumab-FITC and/or adalimumab-Alexa fluor 647
- Quantify the ratio of circulating to resident cells in the intestinal mucosa for each antibody

* If infusion days are staggered, W22 will be assessed two weeks after the 4ème infusion of Vedolizumab.

2.2.2. Secondary endpoint(s)

For all patients:

1. Number of circulating cells binding vedolizumab-FITC and/or adalimumab-Alexa fluor 647 at W0 for all patients
2. Clinical remission at **W22*** defined by a clinical MAYO sub-score ≤ 2 with no individual criteria > 1
3. Clinical response to **W22*** defined by a reduction in the MAYO sub-score of at least 3 points or 30% of the baseline score and a rectal bleeding score of 0 or 1.
4. Endoscopic remission at **W22*** defined by an endoscopic MAYO sub-score of 0 or 1
5. Histological remission at **W22*** defined by a Geboes sub-score < 3.1 (neutrophil infiltrate in the epithelium)

For patients who have failed vedolizumab at W22* and are being treated with second-line adalimumab:

1- Clinical remission at **S30**** defined by a clinical MAYO sub-score ≤ 2 with no individual criteria > 1

2- Clinical response at **S30**** defined by a reduction in the MAYO sub-score of at least 3 points or 30% of the baseline score and with a rectal bleeding score of 0 or 1.

3- Endoscopic remission at **S30**** defined by an endoscopic MAYO sub-score of 0 or 1

4- Histological remission at **S30**** defined by a Geboes sub-score < 3.1 (neutrophil infiltrate in the epithelium)

* If the days of infusion are staggered, W22 will be assessed two weeks after the 4ème infusion of Vedolizumab.

** **If the** days of infusion are staggered, S30 will be assessed two weeks after the 4ème injection of Adalimumab.

2.3. OBJECTIVE AND EVALUATION CRITERIA FOR ANCILLARY STUDIES

Residual concentrations will be analysed as soon as the ELISA assay kits for vedolizumab are marketed.

Objectives:
Quantify residual serum levels of vedolizumab and adalimumab before each infusion/injection to assess the relationship between serum levels and response to treatment.
Assessment criteria :

- For all patients: residual serum vedolizumab levels before each infusion
- Adalimumab serum levels at S30 (or two weeks after the 4ème Adalimumab infusion) for patients who have failed vedolizumab and are being treated with second-line adalimumab.

3. RESEARCH DESIGN

3.1. GENERAL RESEARCH METHODOLOGY

The research has the following characteristics:

- Physiopathology study
- National **multicentre** study
- **Open** study,
- **Prospective** study,

3.2. DIAGRAM OF THE STUDY

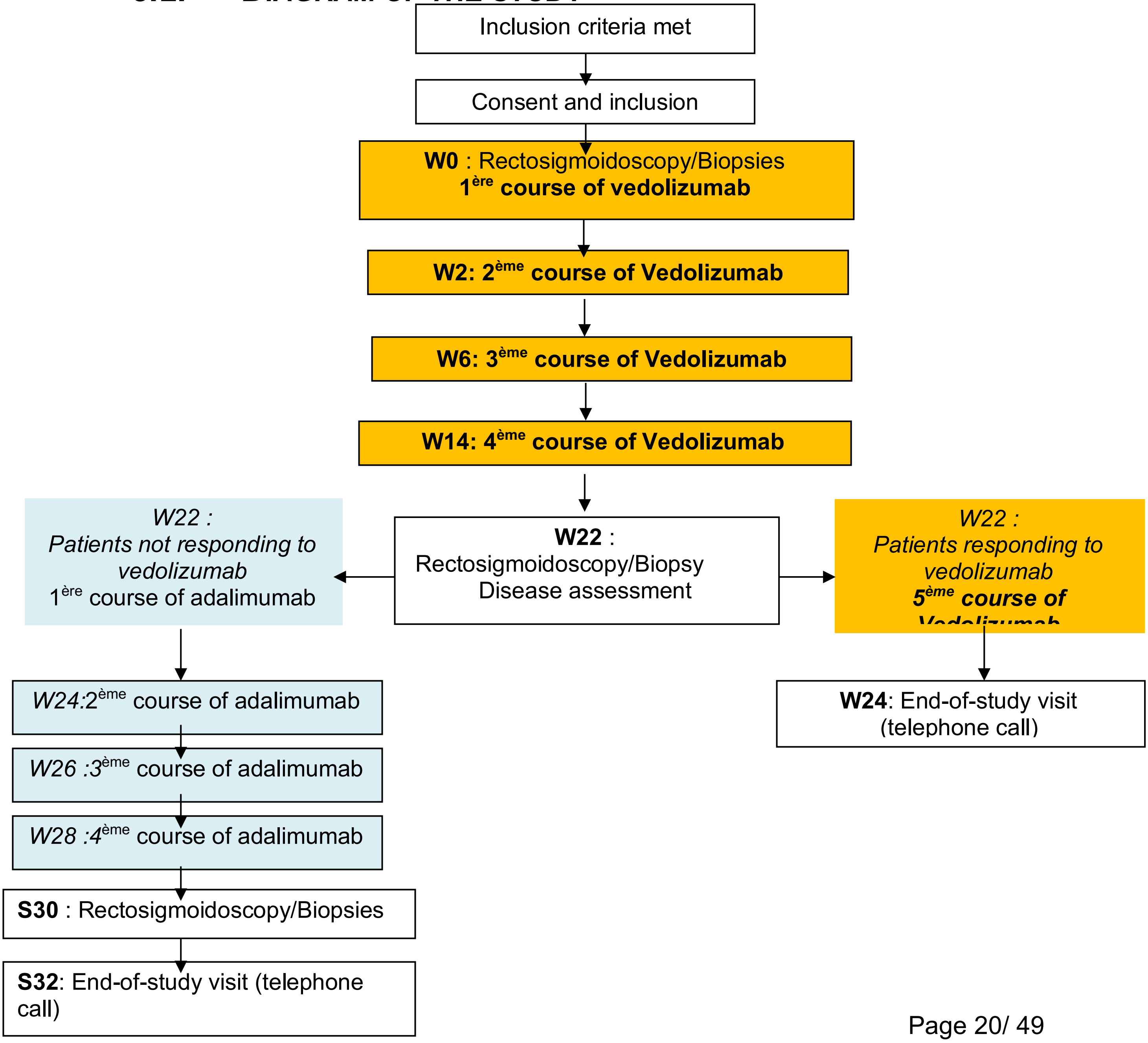

4. POPULATION STUDIED

4.1. DESCRIPTION OF THE POPULATION

This is an exploratory study involving uncertainty about the number of patients to be included. No data have been published on cell labelling with vedolizumab-FITC. No data have been published on the double labelling of vedolizumab-FITC and adalimumab-Alexa fluor 647 or on the analysis of biopsies in contact with these two antibodies by Cellvizio® .
It is planned to include 25 patients with moderate to severe active UC who have not been treated with biotherapies.
The number of patients was assessed to enable statistical analysis of the results, taking into account possible recruitment over the duration of the study in the investigating centres. At Nantes University Hospital, the IMAD sees 10 patients a year with moderate to severe active UC who have not been treated with vedolizumab and adalimumab and who require biotherapy.
The period of exclusion from participation in any other study covers the duration of the study. Participation in non-interventional studies is authorised for the duration of the study.

4.2. INCLUSION CRITERIA

- Patient aged over 18
- Patients with moderate to severe UC defined by an overall MAY0 score ≥ 5 and an endoscopic sub-score ≥ 2 points and a rectal bleeding score ≥ 1 point
- Extension > 15 cm from anal margin
- Requiring treatment with biotherapy after failure of conventional treatments and meeting the indications for treatment with biotherapy
- Affiliated to a social security scheme
- Having signed an informed consent form

4.3. CRITERIA FOR NON-INCLUSION

- Crohn's disease or unclassified colitis
- Severe acute colitis
- Need for immediate surgical treatment
- Previous treatment with vedolizumab or anti-TNF-α
- Contraindication to the use of vedolizumab or an anti-TNF-α agent
- Corticosteroid therapy > 20 mg/d
- Corticosteroid therapy started within the previous 2 weeks
- Conventional immunosuppressant started in the previous month
- Colonic dysplasia or known cancer
- Foreseeable refusal to have two recto-sigmoidoscopies 22 weeks apart
- Severe active infection or opportunistic infection
- Pregnant or breast-feeding women
- Minors
- Adults under guardianship

5. TREATMENTS USED DURING THE STUDY

5.1. DESCRIPTION OF TREATMENTS REQUIRED AND METHODS OF ADMINISTRATION

5.1.1. Investigational medicinal product(s) / comparator

5.1.1.1. Identifying treatments

Vedolizumab (ENTYVIO® 300MG powder for dilute solution for infusion)

- INN: Vedolizumab
- Composition:
  - o Active ingredient: Vedolizumab
  - o Excipients : L-histidine, L-histidine monohydrochloride, L-arginine hydrochloride, sucrose, polysorbate 80
- Manufacturer of the compound and/or marketing authorisation holder: TAKEDA Pharma A/S
- Dosage form and packaging used: powder for concentrated solution for injection. The product has had marketing authorisation in France and the European Community since 22 May 2014.
- Method of administration: intravenous infusion.

After reconstitution, each ml contains 60 mg of Vedolizumab. The dose is 300 mg/infusion at W0, W2, W6 and then every 8 weeks.

5.1.1.2. Administration

Vedolizumab is administered intravenously only. It must be reconstituted and diluted before intravenous administration, in accordance with the Product Monograph. Vedolizumab is administered as an intravenous infusion over 30 minutes.

5.1.1.3. Adjusting dosage

No dosage adjustment is envisaged.

5.1.1.4. *Reference documents*

RCP of ENTYVIO 300MG powder for dilute solution for infusion.

5.1.2. Other medicines in the protocol

5.1.2.1. *Identification of associated treatments*

Adalimumab (*HUMIRA*® 40MG/0.8ML solution for injection in pre-filled pen

- INN: Adalimumab
- Composition:
    - Active substance: adalimumab
    - Excipients : Mannitol, Citric acid monohydrate, Sodium citrate, Monosodium phosphate dihydrate, Disodium phosphate dihydrate, Sodium chloride, Polysorbate 80, Sodium hydroxide, Water for injections.
- Drug manufacturer and/or MAH: AbbVie Ltd
- Dosage form and packaging used: solution for injection in a pre-filled pen.
  The product has had marketing authorisation in France since September 2003.
- *Method of administration:* subcutaneous administration. The dose is *160 mg at W0, 80 mg at W2 and then 40 mg every fortnight.*

5.1.2.2. *Administration*

HUMIRA® is administered subcutaneously, in accordance with the RCP.

5.1.2.3. *Adjusting dosage*

No dosage adjustment is envisaged.

5.2. AUTHORISED AND PROHIBITED MEDICINES AND TREATMENTS

5.2.1. Authorised treatments

All treatments are authorised except those listed in paragraph 5.2.2.

5.2.2. Unauthorised treatments

- Initiation of treatment with corticosteroids and conventional IS
- Optimisation of vedolizumab treatment
- Initiation of an anti-TNF other than adalimumab
- Starting adalimumab before W22
- Initiation of antibiotic or probiotic treatment lasting more than two weeks or indicated for the treatment of UC.
- Topical treatments indicated for UC (5-amino-salycilates, corticosteroids)
- Treatment with corticosteroids (< 20 mg/d) or other treatments started before inclusion will be maintained at a stable dose for the duration of the study.

5.2.3. Emergency treatment

Emergency treatments are those required as part of the patient's usual medical practice or to manage adverse reactions.

5.3. METHODS FOR MONITORING COMPLIANCE WITH TREATMENT

ENTYVIO® will be supplied by the sponsor and dispensed by the pharmacies of the investigating centres, which will monitor the study's therapeutic units (accounting, compliance, etc.) from inclusion to the 5ème injection. It will be returned by the department and destroyed by

the hospital pharmacies after approval by the sponsor. Administration will be traceable in the patient file.

5.4. *INVESTIGATIONAL MEDICINAL PRODUCT CIRCUIT*

5.4.1. General circuit

As part of the study, the laboratory holding the marketing authorisation (TAKEDA) will supply ENTYVIO® to the sponsor at the rate of five injections per patient.
The pharmacy of Hôtel-Dieu, CHU de Nantes, will be the coordinating pharmacy and, as such, will receive the investigational product ENTYVIO® from the TAKEDA laboratories. It will also be responsible for labelling, storing ENTYVIO® and supplying the pharmacies of the investigating centres, in accordance with Good Practice.
Dispensing and destruction operations will be carried out by the site pharmacies, in compliance with the protocol and Good Manufacturing Practices.

5.4.2. Storage conditions for investigational medicinal products

5.4.2.1. Description of storage in the pharmacy

ENTYVIO® will be stored in the pharmacies of the investigating centres in accordance with the conditions defined in the product's SPC.

5.4.2.2. Description of storage in the service

ENTYVIO® will be dispensed by name by the pharmacies of the investigating centres. The department will reconstitute ENTYVIO® in accordance with the RCP, on the same day as it is dispensed.

6. CONDUCT OF THE STUDY

6.1. RESEARCH AND ANALYSIS TECHNIQUES

6.1.1. Detailed description of effectiveness assessment parameters

Assessment of therapeutic response is based on the MAYO clinico-endoscopic score and the Geboes histological score. The evaluation criteria are described below.

- Clinical remission at W22* defined by a clinical MAYO sub-score ≤ 2 with no individual criteria > 1
- Clinical response to W22* defined by a reduction in the MAYO sub-score of at least 3 points or 30% of the baseline score and a rectal bleeding score of 0 or 1.
- Endoscopic remission at W22* defined by an endoscopic MAYO sub-score of 0 or 1
- Histological remission at W22* defined by a Geboes sub-score < 3.1 (neutrophil infiltrate in the epithelium)

For patients who have failed vedolizumab at W22 and are being treated with second-line adalimumab:

- Clinical remission at S30** defined by a clinical MAYO sub-score ≤ 2 with no individual criteria > 1
- Clinical response at S30** defined by a reduction in the MAYO sub-score of at least 3 points or 30% of the baseline score and with a rectal bleeding score of 0 or 1.
- Endoscopic remission at S30** defined by an endoscopic MAYO sub-score of 0 or 1
- Histological remission at S30** defined by a Geboes sub-score < 3.1 (neutrophil infiltrate in the epithelium)

* If the days of infusion are staggered, W22 will be assessed two weeks after the 4ème infusion of Vedolizumab.

** If the days of infusion are staggered, S30 will be assessed two weeks after the 4ème injection of Adalimumab.

6.1.2. Description of techniques and analyses

6.1.2.1. *Rectosigmoidoscopy*

Rectosigmoidoscopy is performed in an endoscopy room in the immediate vicinity of the Gastro-Nutrition CIC and the INSERM U913 unit. It is performed using a video endoscope after preparation with a Normacol® enema administered 2 hours before the examination. The endoscope is advanced under visual control with minimal insufflation guided by the patient's tolerance. The endoscope is advanced to the level of the sigmoid (i.e. approximately 30 - 40 cm above the anal margin).
The entire examination will be recorded on a video medium available in the centres. In the event of a discrepancy between the clinical and endoscopic responses, the recordings will be reviewed by the principal investigator.
During this endoscopy, 8 biopsies will be taken from pathological areas in the sigmoid and, if possible, from healthy areas. 2 biopsies will be analysed in anatomo-pathology to assess microscopic inflammation using the Geboes score and to check for the absence of CMV infection. Six biopsies will be recovered and sent to UMR 913 (Nantes). Three biopsies will be used fresh for an application of vedolizumab coupled to FITC and adalimumab coupled to Alexa fluor 647 (10 minutes at 37°C) and three biopsies will be frozen in nitrogen.
Biopsies are taken using a single-use stingless forceps (Olympus biopsy forceps without needle, ref. FB210K).

Bleeding complications during rectosigmoidoscopy are exceptional (less than 0.02% of procedures). A distinction is made between immediate haemorrhage and delayed haemorrhage. In the case of immediate haemorrhage following a biopsy, endoscopic monitoring enables the evolution of the haemorrhage to be followed, with the possibility of spontaneous cessation or endoscopic haemostasis. In the event of delayed haemorrhage (the delay can vary from a few hours to 21 days), the patient contacts the referring doctor (procedure and telephone number mentioned in the study information letter) who carried out the examination.

6.1.2.2. *Ex vivo experimental design*

6.1.2.3. *Coupling of FITC (495 nm) to vedolizumab and Alexa fluor 647 (647 nm) to adalimumab*

FITC will be covalently conjugated to the humanised IgG1 monoclonal anti-α4β7 antibody (vedolizumab; Entivyo®, Laboratoire Takeda) using specific coupling reagents (Thermo Fisher Scientific, Ref. 53027). Vedolizumab is taken up in boric acid solution (50 mM, pH 8.5) and the concentration is adjusted to 2 $mg.mL^{-1}$. It was then added to 37.5 µg of FITC (96 nmoles) in 0.5 mL of vedolizumab. The sample was mixed and protected from light for 1 hour at 25°C. Excess unbound FITC was removed by eluting the sample on a purification resin. The binding ratio of FITC to vedolizumab is estimated from absorbance measurements of the sample at 280 nm and 495 nm, using the formula: A495/(A280 - 0.32 A495). The concentration of vedolizumab-FITC will be adjusted to 1 $\mu g.\mu L^{-1}$.
The purity of vedolizumab will be checked on an SDS-PAGE gel to exclude the presence of free FITC. After electrophoresis, the gel will be exposed to ultraviolet light to detect fluorescence (Bio-Rad Molecular Imager XRS+ System).
Following the same approach, Alexa fluor 647 will be covalently conjugated to the humanised IgG1 monoclonal anti-TNF-α antibody (adalimumab; Humira®, Abbvie) using specific coupling reagents (Thermo Fisher Scientific, Ref. 53031).

6.1.2.4. *Ex vivo molecular imaging.*

A total of 3 biopsies per patient will be used following the protocol used by Atreya (6). Three biopsies will be rinsed in PBS (37°C) and then incubated with 20 µg vedolizumab-FITC per 500 µL PBS for 10 minutes at 37°C. After rinsing with PBS, the biopsies were analysed by confocal endomicroscopy (Cellvizio®) with two wavelengths, 488 and 660 nm, enabling the two fluorescent markers (FITC and Alexa fluor 647) to be visualised separately on the same sample.

6.1.2.5. *Confirmation of labelling specificity and immunophenotyping of cells labelled with each antibody*

For each patient, 3 labelled biopsies will be cryopreserved for immunofluorescence analysis. Cell nuclei will be immunostained with DAPI and $CD3^+$, $CD8^+$, $CD4^+$ (lymphocytes, dendritic cells) or $CD14^+$ (macrophages, neutrophils or enterocytes) on

7 µm thick sections. Double-positive cells will validate the vedolizumab-FITC labelling. 2 biopsies will be fixed with 4% paraformaldehyde and embedded in paraffin for conventional histological study and immunofluorescence with vedolizumab-FITC to confirm the results obtained previously. Additional staining will be performed with FITC-labelled immunoglobulin G1 (negative control, BD PharMingen), CD14 (dilution 1:50; clone M5E2; BD Biosciences) or CD4 (dilution 1:10; clone RPA-T4; BD Biosciences).

6.1.2.6. Determination of serum biotherapy levels (ancillary study)

Blood samples (10 mL dry tube) will be centrifuged and serum aliquoted into 1 mL cryotubes. The tubes will be stored at -80°C at the Biological Resources Centre of the Nantes University Hospital until they are used. Serum concentrations of vedolizumab will be determined using a plate ELISA technique. Assays will begin as soon as the kits are available.

6.2. STUDY TIMETABLE

Rectosigmoidoscopies (W0, W22) will be performed at the Nantes University Hospital, IMAD. For patients who are non-responders to vedolizumab and are being treated with adalimumab, the rectosigmoidoscopy scheduled at S30 will be performed in each of the investigating centres to which the patient belongs. All injections of biotherapies (vedolizumab and adalimumab) will be administered in each investigating centre.

Screening in investigating centres
During this visit, the inclusion and non-inclusion criteria will be checked. Oral and written information will be given to the patient to explain the objectives and principles of the study.

During the visit, the pre-therapeutic assessment required for the administration of vedolizumab will be prescribed if necessary, the vedolizumab infusion schedule will be drawn up corresponding to the follow-up visits (W0, W2, W6, W14 , W22), and the recto-sigmoidoscopy appointments at the Nantes University Hospital for W0 and W22 will be made.

Visit W0
The inclusion and non-inclusion criteria will be checked again, and the absence of contraindications to the use of vedolizumab will also be verified. During this visit, the patient's consent will be verified before any study procedure is carried out.

During this visit, the clinico-endoscopic MAYO score will be calculated, and recto-sigmoidoscopy will be performed after preparation with a Normacol® enema administered two hours before the examination. Recto-sigmoidoscopy will be performed before the first vedolizumab infusion. A detailed description of the lesions will be made and the videos recorded.

During this endoscopy, 8 biopsies will be taken from pathological areas in the sigmoid and, if possible, from healthy areas. 2 biopsies will be analysed in anatomo-pathology to assess microscopic inflammation using the Geboes score and to check for the absence of CMV infection. Six biopsies will be recovered and sent to UMR 913. Three biopsies will be used fresh for application of vedolizumab coupled to FITC and adalimumab coupled to Alexa fluor 647 (10 minutes at 37°C) and three biopsies will be frozen in nitrogen. After application of the fluorescent antibodies, each biopsy will be examined by confocal endomicroscopy (Cellvizio®) and the quantification of cells positive for each antibody will be recorded by an investigator independent of the patient's clinical and endoscopic follow-up. The three frozen biopsies will be used for immunophenotyping of cells binding the anti α4β7 antibody coupled to FITC and adalimumab coupled to Alexa fluor 647. The markers tested by immunofluorescence will be used to identify cells expressing α4ß7: CD3, CD8, CD4, CD14.

A 10 mL tube of blood will be taken to count cells binding vedolizumab-FITC and/or adalimumab-Alexa fluor 647 and a 10 mL dry tube will be taken to determine serum levels of vedolizumab (indicative baseline value).

At W0, the first infusion of vedolizumab will be carried out in accordance with the investigating centre's usual practice.

Visits W2, W6, W14

During each of these visits, the MAYO clinical subscore will be calculated. A clinical examination will be carried out and the absence of contraindications to vedolizumab infusion will be verified.

A dry 10 mL tube of blood will be taken before each infusion for the determination of residual vedolizumab levels.

In the event of temporary contraindication, the infusion will be postponed and the frequency of follow-up visits will be similarly postponed in order to ensure that all patients receive 4 infusions of vedolizumab before the final analysis.

In the event of intolerance or side effects requiring interruption of Vedolizumab treatment regardless of efficacy, patients will be identified and analysed as failures and will be treated with adalimumab without waiting for W22. Subsequent study visits will be staggered to comply with the protocol intervals.

Visit W22 (or after 4 injections of Vedolizumab)

During this visit, the MAYO clinico-endoscopic score will be calculated, and recto-sigmoidoscopy will be performed after preparation with a Normacol® enema two hours before the examination. A detailed description of the lesions will be made as well as a video recording. Two biopsies will be taken in order to calculate the Geboes histological score.

Patients who respond to Vedolizumab will have a 5ème infusion after recto-sigmoidoscopy at their investigating centre.

Patients who fail vedolizumab will have 6 additional biopsies taken during recto-sigmoidoscopy, which will be recovered and sent to UMR 913. Three biopsies will be used fresh for application of vedolizumab coupled to FITC and adalimumab coupled to Alexa fluor 647 (10 minutes at 37°C) and three biopsies will be frozen in nitrogen. After application of the fluorescent antibodies, each biopsy will be examined by confocal endomicroscopy (Cellvizio®) and the quantification of cells positive for each antibody will be recorded by an investigator independent of the patient's clinical and endoscopic follow-up.

A 10 mL tube of blood will be taken to count cells binding vedolizumab-FITC and/or adalimumab-Alexa fluor 647 in non-responders.

A 10 mL dry tube of blood will be taken prior to infusion for determination of residual vedolizumab levels.

Patients who do not respond to vedolizumab may be treated with adalimumab if there are no contraindications and depending on the decision of the doctor responsible for the patient. Patients will be treated according to the schedule recommended by the AMM (four courses of treatment with one course every 2 weeks: 1ère course of 160 mg at W22 then 80 mg at 2ème course then 40 mg at courses 3 and 4.

Visit W24 (patients responding to vedolizumab at W22)

This visit will be carried out two weeks after the 5ème infusion of Vedolizumab.

Patients considered to be vedolizumab responders by the investigator will have an exit visit 2 weeks later (telephone call) to check the tolerability of the rectosigmoidoscopy performed at W22.

Visit W24 W26 W28: (patients not responding to vedolizumab at W22)

Patients who do not respond to vedolizumab may be treated every fortnight with adalimumab. The first injection of Adalimumab may be given at the investigator's discretion, without waiting for W22.

Visit S30 (non-responders to vedolizumab at W22)

This visit will be carried out two weeks after the 4ème injection of Adalimumab.

During this visit, the MAYO clinico-endoscopic score will be calculated, and recto-sigmoidoscopy will be performed after preparation with a Normacol® enema two hours before the examination. A detailed description of the lesions will be made as well as a video recording. Two biopsies will be taken in order to calculate the Geboes histological score.

A 10 mL dry tube of blood will be taken to determine residual adalimumab levels.

Visit S32 (non-responders to vedolizumab at W22)

Patients considered non-responders to vedolizumab by the investigator will have an exit visit 2 weeks later (telephone call) to check the tolerability of the rectosigmoidoscopy performed at S30.

STUDY TIMETABLE

All patients				Patients responding to Vedolizumab		Patients not responding to Vedolizumab			
**Actions**	Pre-inclusion visit	W0 (Inclusion visit)	W2 W6 W14	W22	W24 End of study	W22	W24 W26 W28	S30	S32 End of study
Patient information	X								
Informed consent	X								
Consent for ancillary pharmacokinetic study	X								
Entering the inclusion in eCRF	X								
History	X								
Clinical examination	X	X	X	X		X		X	
MAYO score (*sub MAYO score)		X	X*	X		X			
Treatment with Vedolizumab		**X**	**X**	**X**					
Treatment with Adalimumab						**X**	**X**		
**Rectosigmoidoscopy (before infusion of biotherapies) with video recording**		X		X		X		X	
2 biopsies for assessment of Geboes Score (pathology department)		X		X		X		X	
6 biopsies for quantification of positive cells and immunophenotyping (UMR913)		X				X			
Blood sample (10mL) for counting biomarker-binding cells		X				X			
Blood sampling (10mL dry tube) for ancillary study		X	X	X		X		X	
Telephone contact to assess the tolerability of the study					X				X
Undesirable events		X	X	X	X	X	X	X	X
Concomitant treatments	X	X	X	X		X	X	X	

6.3. *IDENTIFICATION OF ALL SOURCE DATA NOT CONTAINED IN THE MEDICAL FILE*

The source data not included in the medical record are: number of cells labelled with vedolizumab-FITC and/or adalimumab-Alexa fluor 647 per mucosal analysis field, number of analysis fields per biopsy and per patient, number of circulating blood cells labelled with vedolizumab-FITC and/or adalimumab-Alexa fluor 647, results of immuno-phenotyping of labelled cells, details of Geboes histological score. Video recording of recto-sigmoidoscopy

6.4. *RULES FOR STOPPING A PERSON'S PARTICIPATION*

6.4.1. Criteria for premature termination of a person's participation in research

A patient will be withdrawn from the study due to withdrawal of consent, death of the patient, deviation from the protocol preventing continuation of the study, occurrence of an SAE preventing continuation of the study.
Study withdrawals can only take effect after confirmation by the investigator and the sponsor. Study withdrawals are always final.

6.4.2. Procedures for premature termination of a person's participation in research

The information to be collected is :

- Date of end-of-study visit and type of data to be collected for the end-of-study visit (to be adapted according to the protocol)
- Reason for early discharge
- Follow-up of patients after study discharge in the event of an SAE
  Special care arrangements

For details of how to use data from people who left the study prematurely, please refer to the statistics section.

6.4.3. Criteria for discontinuing all or part of the research (excluding biostatistical considerations)

The end of the research corresponds to the end of the participation of the last person to take part in the research.

Part or all of the study may be stopped permanently or temporarily by decision of the ANSM, the CPP, the Study Sponsor or the CIS (where applicable).
In the event of premature termination of the study at the decision of the Study Sponsor or the CIS (where applicable), the ANSM and the CPP will be informed by letter within a maximum of 15 days.

In all cases :
- A written confirmation will be sent to the study coordinating investigator (specifying the reasons for early termination) as well as to the principal investigator of each centre where applicable,
-All patients in the study will be informed and asked to complete their early discharge visit.
To complete this section, please refer to the "statistics" section, which sets out the statistical criteria for discontinuing research.

6.5. *HOW PATIENTS ARE CARED FOR AT THE END OF RESEARCH*

There is no change in the way patients are managed as a result of their participation in the study. Subsequent management will be decided by the referring doctor in agreement with the patient.
The ANSM Transparency Commission does not plan to reimburse Vedolizumab for first-line treatment.
If a Vedolizumab responder withdraws from the study before receiving the five Vedolizumab infusions specified in the protocol, the sponsor will not pay for any infusions carried out after the patient has withdrawn from the study.
After the last day of protocol treatment (W22 or 5ème infusion of Vedolizumab) the sponsor will not pay for infusions of Vedolizumab.

6.6. *FINAL STUDY REPORT*

The final study report includes a full written description of the research. This report will be sent to the competent authorities and ethics committee less than a year after the end of the study.

The final report is written in collaboration with the sponsor and the study's coordinating investigator.

7. DATA MANAGEMENT AND STATISTICS

7.1. COLLECTION AND PROCESSING OF STUDY DATA

7.1.1. Data collection

An observation book (eCRF) will be created for each patient. All the information required by the protocol must be provided in the eCRF. It must include the data needed to confirm compliance with the protocol and all the data needed for statistical analysis; it must also enable major deviations from the protocol to be identified.

The person(s) responsible for filling in the eCRFs (investigator, CRA, etc.) must be defined and is/are identified in each centre's table of delegated responsibilities (kept in the investigator's folder).

7.1.2. Data coding

By signing this protocol, the principal investigator and all co-investigators undertake to maintain the confidentiality of the identities of the patients taking part in the study.

The anonymity of the subjects will be ensured by mentioning the first letter of the surname and the first letter of the first name on all documents.
This code will be the only information to appear in the observation book (eCRF) and will enable the eCRF to be linked to the patient at a later date.
The investigator is also required to code patient data on any documents in his possession (reports of imaging or biological examinations, etc.) which are attached to the eCRF.

7.1.3. Data processing

The collection of clinical data will be based on the creation of a database and data entry masks similar to the observation notebook, in compliance with the protocol and regulations currently in force.
The structure of the database and input screens will be approved by the trial sponsor.
At the end of the study, the CRF database is reconciled with the safety database. This reconciliation is carried out before the database is frozen. Similarly, an annual reconciliation is carried out when the Annual Safety Report (ASR) is updated.

7.2. STATISTICS

Clinical and demographic data will be collected at study entry: DOB (month/year), date of diagnosis, disease phenotype (extension), family history of IBD, personal history of surgery, smoking status, treatment at study entry, history of corticosteroid use (yes/no), history of immunosuppressive use (yes/no), clinical symptoms and endoscopic evaluation for calculation of the MAYO score. Clinical data to calculate the MAYO score will be collected at each follow-up visit. Endoscopic evaluation with lesion description and video recording is planned at inclusion and at W22 ± S30 for non-responders to vedolizumab and treated with adalimumab as second-line therapy. Histological data (Geboes score) will be collected during endoscopic assessments. Treatment tolerance will be assessed at each visit and 2 weeks after the end of the study by telephone call.

7.2.1. Description of planned statistical methods, including timetable for planned interim analyses

Test performance
A sensitivity/specificity analysis (ROC curve) will be performed to identify the optimal threshold of labelled cells per field and thus classify patients into a vedolizumab responder and non-responder subgroup at W22 (two weeks after the 4ème infusion of Vedolizumab) based on the results of the MAYO clinico-endoscopic score.

Data analysis

Clinical and demographic data on patients will be collected at the inclusion visit and at follow-up visits. All included patients will be retained for final analysis (ITT) and all patients who have received at least one dose of study product will also be analysed (modified intent to treat population).
Quantitative data will be expressed as mean ± standard deviation or median (interquartile range) according to the likelihood of the normality of their distribution (attested by a Kolmogorov-Smirnov test), while qualitative data will be expressed in the form of numbers and corresponding percentages.
Descriptive analyses will be carried out for all the variables collected, and point estimates and 95% confidence intervals will be calculated for the qualitative and quantitative variables.
Comparisons and analyses of associations between variables will be carried out, depending on the quantitative or categorical nature of the variables explored, their distribution and the corresponding numbers, using parametric or non-parametric tests, chi-square or Fisher tests; logistic regression models will be used to estimate the relationship between the number of cells labelled with vedolizumab and the remission, clinical and/or endoscopic and histological response rates at week 22 (two weeks after the 4ème infusion of Vedolizumab). Odds ratios and 95% confidence intervals will be estimated. The goodness-of-fit of the models will be attested by the Hosmer-Lemeshow test and their predictive capacity will be assessed using the estimated area under the ROC curve.

7.2.2. Statistical justification of the number of inclusions

This is an exploratory study involving uncertainty about the number of patients to be included. No data have been published on cell labelling with vedolizumab-FITC. No data have been published concerning double labelling with vedolizumab-FITC and adalimumab-Alexa fluor 647. No data has been published on the analysis of biopsies in contact with these two antibodies by Cellvizio®. The number of patients (25) was evaluated to allow statistical analysis of the results, taking into account possible recruitment over the duration of the study in the investigating centres.

7.2.3. Expected level of statistical significance

The significance level will be 5%.

7.2.4. Statistical criteria for discontinuing research

Not applicable

7.2.5. Method for taking into account missing, unused or invalid data

Patients in whom vedolizumab should be discontinued because of poor tolerability despite clinical efficacy will be identified. They will be considered ITT and mITT vedolizumab failures. An additional post-hoc analysis may be performed based on the efficacy of the treatment independently of side-effects and will be clearly identified in the results.
The number of patients lost to follow-up is expected to be limited given the treatment modalities and hyper-selective recruitment. Patients lost to follow-up who received at least one infusion of vedolizumab will be included in the final analysis (mITT).

7.2.6. Managing changes to the initial strategy analysis plan

The initial analysis plan will not be modified.

7.2.7. Choosing the people to be included in the analyses

All included patients will be retained for the final analysis (ITT) and all patients who have received at least one dose of study product will also be analysed (modified intent to treat population).

8. PHARMACOVIGILANCE AND ADVERSE EVENT MANAGEMENT

8.1. DEFINITIONS

Vigilance	This is the monitoring of medicines, medical devices and other health products. It also involves preventing the risk of undesirable effects resulting from their use, whether this risk is potential or proven,
Adverse events	Any harmful event in a patient or participant in a clinical trial, not necessarily related to the treatment planned in the clinical trial.
Intensity of adverse events (AE)	It will be graded according to the criteria chosen when the protocol was drawn up. For any event not noted in the chosen classification, the rating will be as follows:<br><br>1 = benign<br>2 = moderate<br>3 = severe<br>4 = life-threatening
Undesirable effects (AE)	Any adverse event for which a causal link, whatever its importance (doubtful, plausible, possible, certain) can be envisaged either with the treatment under study or with the comparator or protocol.
Serious adverse events (SAEs)/Evènements indésirables graves (EvIG)	Any adverse reaction/event that :<br>* results in death,<br>* is life-threatening,<br>* results in temporary or permanent incapacity or disability,<br>* requires or prolongs the patient's hospitalisation,<br>* causes a congenital or neonatal anomaly,<br>* is medically important (the list of medically important effects/events is defined by the EMA).

Unexpected adverse events (AEIs)	An effect whose nature, severity, frequency or course are not consistent with the product information as listed in the investigator's brochure, the Summary of Product Characteristics or as defined in the protocol.
Overdose	Actual= Administration of the medicinal product in a quantity greater (in a single or cumulative administration) than that authorised in the Summary of Product Characteristics, the investigator's brochure.<br>Relative= Administration of the drug at the recommended dosage but not adapted to the person (malnutrition, dehydration, renal failure, etc.).
Misuse	Inappropriate use in relation to relevant reference data, occurring during the care chain, exposing a given patient to a proven or potential risk, without any correlative benefit
Medication errors	Corresponds to the omission or unintentional performance of an act during the care process involving a medicinal product, which may result in a risk or adverse event for the patient.

8.2. SECURITY ASSESSMENT PARAMETERS

8.2.1. Specific safety-related assessment criteria

Not applicable

8.2.2. Methods and timetable for measuring, collecting and analysing safety assessment parameters

AEs and SAEs will be investigated by the investigating physicians. AEs and MAEs will be recorded in the CRF. The information collected will include the dates of onset of symptoms, description of symptoms, evolution, measures taken with regard to the patient and the medical device, biological tests and additional examinations undertaken.

8.3. Reports will be analysed by the pharmacovigilance department, which will summarise them. *List of expected ARs*

1. Related to the specific examinations in the protocol :

Rectosigmoidoscopy at W0 and W22 is a routine procedure.

The expected ARs are :
- abdominal bloating,
- spastic pain during the examination;
- Other complications are possible but exceptional (bleeding, digestive discomfort and temporary leaks).

2. Pathology-related :

Worsening of symptoms: diarrhoea, rectal discharge, abdominal pain, asthenia, fever, venous thrombosis, etc.

3. Treatment-related (vedolizumab and adalimumab) :

The drugs used will be within the scope of their MA. The expected AEs are listed in the summaries of product characteristics (SPCs) for the treatments.
The reference document will be the SPC for Entyvio® dated 11/01/2016 or, if applicable, its revised version.
For associated and concomitant drugs, the documents will be the SPCs in force on the date of the AR.
Only AEs related to specific examinations will be recorded in the CRF.

8.4. MANAGING UNDESIRABLE EVENTS

8.4.1. Notification of SAEs / EvIG

In the context of the protocol, only SAEs/STEEs related to rectosigmoidoscopy and biopsies require the completion of a SAE/STEE report, whether expected or not. The investigator must check that the information given on this form is complete, accurate and clear (do not use abbreviations, etc.). The SAE report and the documents attached to this report must be coded.
The SAE / SAE must be reported immediately (within 24 hours of its detection by the investigator) to the sponsor.
After receiving notification of an unexpected adverse event, the sponsor declares it to the regulatory authorities. Once a year, it draws up an annual safety report.
NB: Pregnancy, overdose, misuse, medication errors or risk of medication errors, and quality defects must be reported to the sponsor by the investigator, even if there is no adverse reaction.

8.4.2. Independent Supervisory Committee

No independent supervisory committee will be set up.

8.5. METHODS AND DURATION OF FOLLOW-UP FOR PEOPLE FOLLOWING THE OCCURRENCE OF UNDESIRABLE EVENTS

All events must be monitored until recovery, consolidation or death (closed event).

9. ADMINISTRATIVE AND REGULATORY ASPECTS

9.1. RIGHT OF ACCESS TO SOURCE DATA AND DOCUMENTS

Each patient's medical data will be transmitted only to the promoter or any person duly authorised by the promoter and, where applicable, to the authorised health authorities, under conditions guaranteeing confidentiality.
The sponsor and the regulatory authorities may request direct access to the medical file in order to verify the procedures and/or data of the clinical trial and within the limits authorised by the laws and regulations.
The data collected during the trial will be processed electronically, in compliance with CNIL requirements (compliance with reference methodology MR001).

9.2. MONITORING THE TRIAL

Monitoring will be carried out by the Promotion Department of the Research Division. A Clinical Research Associate (CRA) will regularly visit each site (investigator and pharmacy) to check the quality of the data reported in the case report forms.

The protocol has been classified according to the estimated level of risk for the patient undergoing the research. It will be monitored as follows:

Risk B: foreseeable risk close to that of usual care

On-site monitoring visits will be organised after making an appointment with the investigator. The CRAs must have access to each site:
- data collection books for the patients included,
- patient medical and nursing records,
- the investigator binder.

9.3. *INSPECTION / AUDIT*

As part of this study, an inspection or audit may take place. The sponsor and/or the participating centres must be able to give the inspectors or auditors access to the data.

9.4. *ETHICAL CONSIDERATIONS*

9.4.1. Written informed consent

The investigator undertakes to inform the patient clearly and fairly about the protocol and to ask for informed written consent (information note and consent form attached). The investigator will give the patient a copy of the information note and a consent form. The patient can only be included in the study after having read the information note and signed and dated the consent form after a period of reflection. The investigator must also sign and date the consent form. A minimum of 2 copies of these two documents must be issued on paper, so that the patient and the investigator can each keep a copy. The investigator's original will be filed in the investigator's folder. In the case of duplicate signed consents, the investigator keeps the original and the duplicate is given to the patient.

9.4.2. Procedures for obtaining consent in the event of an emergency

Not applicable

9.4.3. Individual Protection Committee

The sponsor undertakes to submit the study project for prior authorisation by a Personal Protection Committee (CPP). The information provided covers both the nature of the research and the safeguards provided for patients taking part in the trial.

9.5. AMENDMENTS TO THE PROTOCOL

Requests for substantial modifications will be submitted by the sponsor for authorisation or information to the ANSM and/or the relevant personal data protection committee in accordance with Law 2004-806 of 9 August 2004 and its implementing decrees.
An updated version of the amended protocol must be dated.
The patient information and consent forms will be amended if necessary.

9.6. DECLARATION TO THE COMPETENT AUTHORITIES

This protocol will be the subject of an application for authorisation to the ANSM.

9.7. REGISTER OF PERSONS UNDERGOING BIOMEDICAL RESEARCH

Not applicable

9.8. FINANCING AND INSURANCE

The sponsor will finance the study and take out an insurance policy covering the financial consequences of its civil liability, in accordance with regulations.

9.9. PUBLICATION RULES

A copy of the publication will be sent to the Nantes University Hospital, the study's promoter, which will necessarily be cited. A copy of the publications will be sent to the industrial partners (Takeda, Mauna Kea). Authorship will be determined in *proportion to* the number of patients included. All centres will be represented among the signatories by one or more authors. The investigator coordinating the study will be cited last and will draw up the list of authors.

As part of the project is financed by calls for projects from the French Ministry of Health, publications will bear the following mention: "This study was supported by a grant from the French Ministry of Health (programme acronym, programme year, registration number: e.g. PHRC 2014 XXXX, or PREPS 2014 XXXX, ...)" and will mention the partnership with Takeda and Mauna Kea.

9.10. FATE OF BIOLOGICAL SAMPLES

At the end of the research, the biological samples resulting from the collection of biopsies and blood will be stored in the IMAD biocollection at Nantes University Hospital, under the responsibility of Dr A Bourreille. This biocollection and the procedure for obtaining consent have been declared to the Ministry of Research under number DC-2008-402.

9.11. ARCHIVING SOURCE DATA

The investigator must keep all information relating to the study for at least 15 years after the end of the study.
At the end of the study, the investigator will also receive a copy of the data for each patient in his centre via a CD-ROM sent by the sponsor.

LIST OF APPENDICES

- Appendix 1: List of investigators
- Appendix 2: Summary of the protocol
- Appendix 3: Bibliographical references
- *Appendix 4: Mayo score*
- Appendix 5: Geboes score
- Appendix 6: Patient information letter
- Appendix 7: Patient consent form
- Appendix 8: RCP VEDOLIZUMAB

APPENDIX 1: LIST OF INVESTIGATORS

SURNAME AND FIRST NAME	Speciality	Function	Name of establishment	Name and address of parent department	Telephone, fax and e-mail	RPPS NUMBER
Bourreille Arnaud	Hepato-gastro-enterology	Hospital practitioner PU	Nantes University Hospital	IMAD<br>Nantes University Hospital<br>1 PLACE ALEXIS RICORDEAU<br>44093 Nantes	Tel : 02 40 08 31 52<br>Fax: 02 40 08 31 54<br><br>Arnaud.bourreille@chu-nantes.fr	10002577517
DIB Nina	Hepato-gastro-enterology	Hospital practitioner	Angers University Hospital	Hepato-gastro-enterology department<br>Angers University Hospital<br>4 Rue Larrey<br>49933 Angers Cedex 09	Tel : 02 41 35 31 43<br>Fax: 02 41 35 36 61<br><br>nidib@chu-angers.fr	10002593969
BOUGUEN Guillaume	Hepato-gastro-enterology	Hospital practitioner	CHRU Pontchaillou	Digestive Tract Diseases Department<br>CHRU Pontchaillou.<br>2, Rue Henri. Le Guilloux. F 35033 Rennes Cedex 9	Tel: 02.99.28.99.72<br>guillaume.bouguen@chu-rennes.fr	10100058436
AMIL Morgane	Gastroenterology	Hospital practitioner	CHD Vendée	Gastroenterology Department<br>CHD Vendée<br>Boulevard Stéphane Moreau<br>85925 LA ROCHE SUR YON	Tel : 02 51 44 61 68<br>Fax: 02 51 44 62 99<br>Morgane.amil@chd-vendee.fr	10100169670

APPENDIX 2: SUMMARY OF THE PROTOCOL

**Title of the study**	**Development of an efficacy biomarker for vedolizumab (Entyvio®) in ulcerative colitis (UC)**
**Key words**	Haemorrhagic rectocolitis; biomarker; vedolizumab; confocal endomicroscopy ;
**Study sponsor**	**NANTES University Hospital**
**Co-ordinating investigator (if multicentre study)**	Prof. Arnaud Bourreille<br>Institute of Digestive System Diseases<br>Nantes University Hospital
**Number of centres planned**	4
**Type of study**	RBM Médicament
**Study schedule**	❖ Total duration: 26 months<br>❖ Recruitment period: 18 months<br>❖ Duration of treatment per patient: maximum 30 weeks*.<br>❖ Follow-up time per patient: 24 to 32 weeks*.<br>*Patients whose treatment days have been postponed will be monitored for up to two weeks after stopping treatment with Vedolizumab or Adalimumab.
**Study design**	❖ multicentre<br>❖ uncontrolled<br>❖ open<br>❖ Foresight
**Aims of the study**	Main objective:<br><br>To demonstrate the feasibility of ex vivo labelling of intestinal immune cells using a combination of two markers : vedolizumab-FITC and adalimumab-Alexa fluor 647 quantifiable at the same time and on the same sample by confocal endomicroscopy using a Cellvizio® probe with two wavelengths (488 and 660 nm) on intestinal biopsies from patients with moderate to severe active UC, to develop a biomarker for the efficacy of vedolizumab.<br><br>Secondary objective(s) :<br><br>To quantify, ex vivo, the number of FITC-coupled vedolizumab-labelled cells in the intestinal mucosa of patients with moderate to severe UC associated with clinical remission at W22* after initiation of vedolizumab treatment.<br>To assess the association between the number of cells labelled with fluorescent vedolizumab and the percentage of clinical remission, clinical response, endoscopic remission and histological remission at W22* after initiation of treatment with standard-dose vedolizumab compared with patients with no or low numbers of positive cells.<br>Compare the number of immune cells positive for each of the fluorescent antibodies in the intestinal mucosa of UC patients.

	Assess the number of cells doubly labelled with the two fluorescent antibodies<br>To evaluate the association between the adalimumab biomarker coupled to Alexa fluor 647 quantified at inclusion and at W22* and the rate of clinical response, clinical remission, endoscopic remission and histological remission to adalimumab in second-line treatment after failure of vedolizumab.<br>Immunofluorescence determination of the cellular phenotype of cells labelled with vedolizumab-FITC and/or adalimumab-Alexa fluor 647<br>Quantify residual serum levels of vedolizumab and adalimumab before each infusion/injection to assess the relationship between serum levels and response to treatment.<br>Quantify the ratio of circulating to resident cells in the intestinal mucosa for each antibody<br>* If the days of infusion are staggered, W22 will be assessed two weeks after the 4th infusion of Vedolizumab.
**Estimated number of cases**	25
**Schedule of visits and examinations**	Visits: Screening, weeks 0, 2, 6, 14, 22 and 30 (initial non-responders)<br>Rectosigmoidoscopy: weeks 0, 22 and 30 (initial non-responders)
**Main selection, inclusion, non-inclusion and exclusion criteria**	Patients with moderate to severe UC defined by an overall MAY0 score ≥ 5 and an endoscopic sub-score ≥ 2 points and a rectal bleeding score ≥ 1 point<br>Requiring treatment with a biotherapy that has failed conventional treatments and meets the indications for treatment<br>Without severe acute colitis or need for immediate surgical treatment, naive to vedolizumab or anti-TNF-α<br>Having signed an informed consent form
**Treatment, interventional procedure under study**	Initial treatment with vedolizumab in accordance with the terms of the marketing authorisation, preceded by outpatient recto-sigmoidoscopy at Nantes University Hospital, during which 6 biopsies will be taken for biomarker identification in the research laboratory.<br>Clinical and endoscopic evaluation at week 22 by 2ème recto-sigmoidoscopy.<br>Treatment with adalimumab according to marketing authorisation in patients who have failed vedolizumab and clinical and endoscopic assessment at week 30
**Primary endpoint**	Number of fluorescent cells per field of examination in cellvizio® for each antibody: vedolizumab coupled to FITC and adalimumab coupled to Alexa fluor 647 at W0 for all patients and at W22 for non-responders to vedolizumab treated with adalimumab.
**Secondary endpoint(s)**	Number of circulating cells binding vedolizumab-FITC and/or adalimumab-Alexa fluor 647 at W0 for all patients and at W22* for vedolizumab non-responders treated with adalimumab<br>Clinical remission at W22* defined by a clinical MAYO sub-score ≤ 2 with no individual criteria > 1<br>Clinical response to W22* defined by a reduction in the MAYO sub-score of at least 3 points or 30% of the baseline score and a rectal bleeding score of 0 or 1.

	Endoscopic remission at W22* defined by an endoscopic MAYO sub-score of 0 or 1<br>Histological remission at W22* defined by a Geboes sub-score < 3.1 (neutrophil infiltrate in the epithelium)<br>Residual serum levels of vedolizumab before each infusion<br>**For patients who have failed vedolizumab at W22 and are being treated with second-line adalimumab:**<br>Clinical remission at S30** defined by a clinical MAYO sub-score ≤ 2 with no individual criteria > 1<br>Clinical response at S30** defined by a reduction in the MAYO sub-score of at least 3 points or 30% of the baseline score and with a rectal bleeding score of 0 or 1.<br>Endoscopic remission at S30** defined by an endoscopic MAYO sub-score of 0 or 1<br>Histological remission at S30** defined by a Geboes sub-score < 3.1 (neutrophil infiltrate in the epithelium)<br>Serum adalimumab levels at S30**<br>* If the days of infusion are staggered, W22 will be assessed two weeks after the 4th infusion of Vedolizumab.<br>** If the days of infusion are staggered, S30 will be assessed two weeks after the 4th injection of Adalimumab.
☐ **Pharmacogenetics**<br>☒ **Pharmacokinetics**<br>☐ **Pharmacodynamics**<br>☐ **Pharmacoeconomics**<br>☐ **Other analyses**	
**Statistical analysis**	A sensitivity/specificity analysis (ROC curve) will be carried out to identify the optimal threshold of labelled cells per field and thus classify patients into responder and non-responder subgroups to vedolizumab at W22*.

APPENDIX 3: BIBLIOGRAPHICAL REFERENCES

1. Rungoe C, Langholz E, Andersson M, Basit S, Nielsen NM, Wohlfahrt J, Jess T. Changes in medical treatment and surgery rates in inflammatory bowel disease: a nationwide cohort study 1979-2011. Gut 2014;63:1607-16.
2. Feagan BG, Macdonald JK. Oral 5-aminosalicylic acid for induction of remission in ulcerative colitis. Cochrane Database Syst Rev 2012;10:CD000543.
3. Feagan BG, Macdonald JK. Oral 5-aminosalicylic acid for maintenance of remission in ulcerative colitis. Cochrane Database Syst Rev 2012;10:CD000544. doi: 10.1002/14651858.CD000544.
4. Laharie D, Bourreille A, Branche J, Allez M, Bouhnik Y, Filippi J, Zerbib F, Savoye G, Nachury M, Moreau J, Delchier JC, Cosnes J, Ricart E, Dewit O, Lopez-Sanroman A, Dupas JL, Carbonnel F, Bommelaer G, Coffin B, Roblin X, Van Assche G, Esteve M, Färkkilä M, Gisbert JP, Marteau P, Nahon S, de Vos M, Franchimont D, Mary JY, Colombel JF, Lémann M; Groupe d'Etudes Thérapeutiques des Affections Inflammatoires Digestives. Ciclosporin versus infliximab in patients with severe ulcerative colitis refractory to intravenous steroids: a parallel, open-label randomised controlled trial. Lancet 2012;380:1909-15.
5. Sandborn WJ, Feagan BG, Rutgeerts P, Hanauer S, Colombel JF, Sands BE, Lukas M, Fedorak RN, Lee S, Bressler B, Fox I, Rosario M, Sankoh S, Xu J, Stephens K, Milch C, Parikh A; GEMINI 2 Study Group. Vedolizumab as induction and maintenance therapy for Crohn's disease. N Engl J Med 2013;369:711-21.
6. Atreya R, Neumann H, Neufert C, Waldner MJ, Billmeier U, Zopf Y, Willma M, App C, Münster T, Kessler H, Maas S, Gebhardt B, Heimke-Brinck R, Reuter E, Dörje F, Rau TT, Uter W, Wang TD, Kiesslich R, Vieth M, Hannappel E, Neurath MF. In vivo imaging using fluorescent antibodies to tumor necrosis factor predicts therapeutic response in Crohn's disease. Nat Med 2014;20:313-8.
7. Vermeire S, O'Byrne S, Keir M, Williams M, Lu TT, Mansfield JC, Lamb CA, Feagan BG, Panes J, Salas A, Baumgart DC, Schreiber S, Dotan I, Sandborn WJ, Tew GW, Luca D, Tang MT, Diehl L, Eastham-Anderson J, De Hertogh G, Perrier C, Egen JG, Kirby JA, van Assche G, Rutgeerts P. Etrolizumab as induction therapy for ulcerative colitis: a randomised, controlled, phase 2 trial. Lancet 2014;384:309-18.

APPENDIX 4: MAYO SCORE

Frequency of bowel movements

Normal	0
1-2 more programmes than usual	1
3-4 programmes more than usual	2
> 4 more programmes than usual	3

Chest pain

Absent	0
Traces of blood	1
Frank bleeding	2
Almost pure blood	3

Mucous membrane

Normal	0
Erythema, reduction in the vascular network, fragility, granular appearance	1
Frank erythema, disappearance of the vascular network, erosions, pus	2
Ulcerations, spontaneous bleeding, pus	3

Overall assessment by the doctor

No active disease	0
Minor illness	1
Moderate illness	2
Severe illness	3

TOTAL = ...

Disease activity :
< 3 remission
4-5 minors
6-9 moderate
9-12 severe

Rutgeerts P, et al. N Engl J Med. 2005; 353: 2462-7.

APPENDIX 5: *GEBOES SCORE*

**Grade 0 Structural (architectural changes)**
Subgrades
0.0 No abnormality<br>0.1 Mild abnormality<br>0.2 Mild or moderate diffuse or multifocal abnormalities<br>0.3 Severe diffuse or multifocal abnormalities
**Grade 1 Chronic infl ammatory infi ltrate**
Subgrades
1.0 No increase<br>1.1 Mild but unequivocal increase<br>1.2 Moderate increase<br>1.3 Marked increase
**Grade 2 Lamina propria neutrophils and eosinophils**
2A Eosinophils
2A.0 No increase<br>2A.1 Mild but unequivocal increase<br>2A.2 Moderate increase<br>2A.3 Marked increase
2B Neutrophils
2B.0 No increase<br>2B.1 Mild but unequivocal increase<br>2B.2 Moderate increase<br>2B.3 Marked increase
**Grade 3 Neutrophils in epithelium**
Subgrades
3.0 None<br>3.1 < 5 % Crypts involved<br>3.2 < 50% Crypts involved<br>3.3 > 50% Crypts involved
**Grade 4 Crypt destruction**
Subgrades
4.0 None<br>4.1 Probable - local excess of neutrophils in part of crypt<br>4.2 Probable - marked attenuation<br>4.3 Unequivocal crypt destruction
**Grade 5 Erosion or ulceration**
Subgrades
5.0 No erosion, ulceration, or granulation tissue<br>5.1 Recovering epithelium + adjacent infl ammation<br>5.2 Probable erosion focally stripped<br>5.3 Unequivocal erosion<br>5.4 Ulcer or granulation tissue

Bessissow et al. The American Journal of Gastroenterology 107, 1684-1692 (November 2012) | doi:10.1038/ajg.2012.301
